# Large Language Model‐Informed Dual‐Track AI Framework for the Synergistic Design of Crack‐Free and High‐Strength Superalloys

**DOI:** 10.1002/advs.76036

**Published:** 2026-06-09

**Authors:** Jian Yao, Junkang Wu, Jie Su, Fuzhu Wang, Zi Wang, Li Wang, Liming Tan, Lan Huang, Feng Liu, Yong Liu

**Affiliations:** ^1^ State Key Laboratory of Powder Metallurgy Central South University Changsha China

**Keywords:** computer science, heuristic, heuristics, materials science, process optimization, pruning, reinforcement learning, superalloy, ultimate tensile strength

## Abstract

Laser Powder Bed Fusion of high performance nickel‐based superalloys is a transformative technology hindered by acute hot‐cracking susceptibility and prohibitively expensive process optimization cycles. Traditional data‐driven AI models often suffer from “black‐box” limitations and severe data scarcity. Here, we propose a knowledge‐informed hybrid AI framework that integrates Large Language Models (LLMs) as reasoning agents to bridge metallurgical expertise with autonomous discovery. By operationalizing empirical heuristics (e.g., Al/Ti ratio correlations), the LLM facilitated rapid heuristic pruning, compressing the initial search space of 76 800 candidates by >95%. Subsequently, LLM‐distilled process priors were injected into a reinforcement learning (RL) agent, enabling a “warm‐start” optimization that achieved safety‐constrained exploration in physically risky regimes. The resulting AMN01 alloy achieved crack‐free printability with a breakthrough yield strength exceeding 1.5 GPa and an ultimate tensile strength near 1.8 GPa in the direct‐aged state. Following solution‐aging treatment, the alloy maintained a UTS over 1.6 GPa with an exceptional elongation of over 15%. Multi‐scale characterization revealed a multi‐tier strengthening architecture involving nanoscale cellular dislocation networks, γ' precipitation, and deformation‐induced Lomer–Cottrell locks. This framework establishes a generalizable paradigm for accelerated material discovery in high‐cost, data‐scarce engineering environments.

## Graphical abstract

This paper illustrates a knowledge‐augmented dual‐track AI framework for advanced superalloy design. First, Large Language Models translate metallurgical heuristics into explicit rules to rapidly prune a vast compositional search space. Subsequently, LLM‐distilled priors safely guide a reinforcement learning agent during autonomous process optimization, successfully yielding AMN01, an ultra‐high‐performance, crack‐free superalloy.

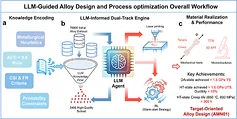

## 1 Introduction

The development of high‐performance nickel‐based superalloys for extreme environments [1], such as aero‐engines and land‐based turbines [2], is critical for enhancing thermodynamic efficiency and reducing carbon emissions. Traditionally, these components are fabricated via investment casting or forging [3, 4, 5]; however, the emergence of Laser Powder Bed Fusion (LPBF) has revolutionized the field by enabling the manufacturing of complex [6], lightweight structures with intricate internal cooling channels that are otherwise unachievable via conventional subtractive methods. Despite these advantages, the unique thermal history of LPBF, characterized by rapid solidification [7] and repetitive thermal cycling, imposes severe challenges. Most high‐performance superalloys designed for casting are highly susceptible to microcracking [8] under AM conditions. Consequently, the design space is not only vast and highly nonlinear [9, 10] but also constrained by the complex interplay between non‐equilibrium solidification and mechanical response [11]. Traditional trial‐and‐error approaches are prohibitively slow, while conventional data‐driven machine learning models often suffer from a “black‐box” nature, leading to alloys that are “digitally optimal” but ignore the fundamental metallurgical constraints of additive manufacturing.

Recent advancements in materials informatics have sought to accelerate alloy development via machine learning (ML) and high‐throughput screening [12, 13, 14, 15, 16]. However, the application of data‐driven models to the complex LPBF process remains constrained by two fundamental bottlenecks. First, the high cost of additive manufacturing experiments leads to severe data scarcity, where sparse and non‐uniform datasets often fail to capture the multi‐dimensional correlations between non‐equilibrium solidification and mechanical response. Second, autonomous process optimization via Reinforcement Learning (RL) suffers from the “cold start” problem. Vanilla RL agents, starting with zero prior knowledge, frequently explore physically non‐viable parameter spaces, resulting in catastrophic trial failures (e.g., severe cracking or mechanical damage). These limitations necessitate a transition from pure connectionist AI to a more sophisticated paradigm capable of reasoning with existing metallurgical logic.

The emergence of Large Language Models (LLMs) offers a transformative “knowledge bridge” to bridge the gap between empirical metallurgical heuristics and numerical optimization. Unlike traditional ML, LLMs possess the unique capacity for symbolic reasoning, enabling the operationalization of unstructured human expertise, such as the “Al/Ti ratio < 0.6” rule for crack suppression, into structured algorithmic constraints. By acting as a reasoning agent, the LLM can perform high‐level “heuristic pruning” on vast compositional spaces, filtering out high‐risk candidates before costly computational or experimental iterations. Crucially, this LLM‐informed approach enables a “warm start” strategy for RL‐driven process optimization. By injecting distilled process heuristics as a prior policy, the agent inherits a form of “metallurgical intuition,” allowing it to bypass physically unviable regimes and converge toward the optimal processing window with unprecedented efficiency. This synergy of symbolic knowledge and autonomous exploration represents a new frontier in intelligent materials design.

**FIGURE 1:**
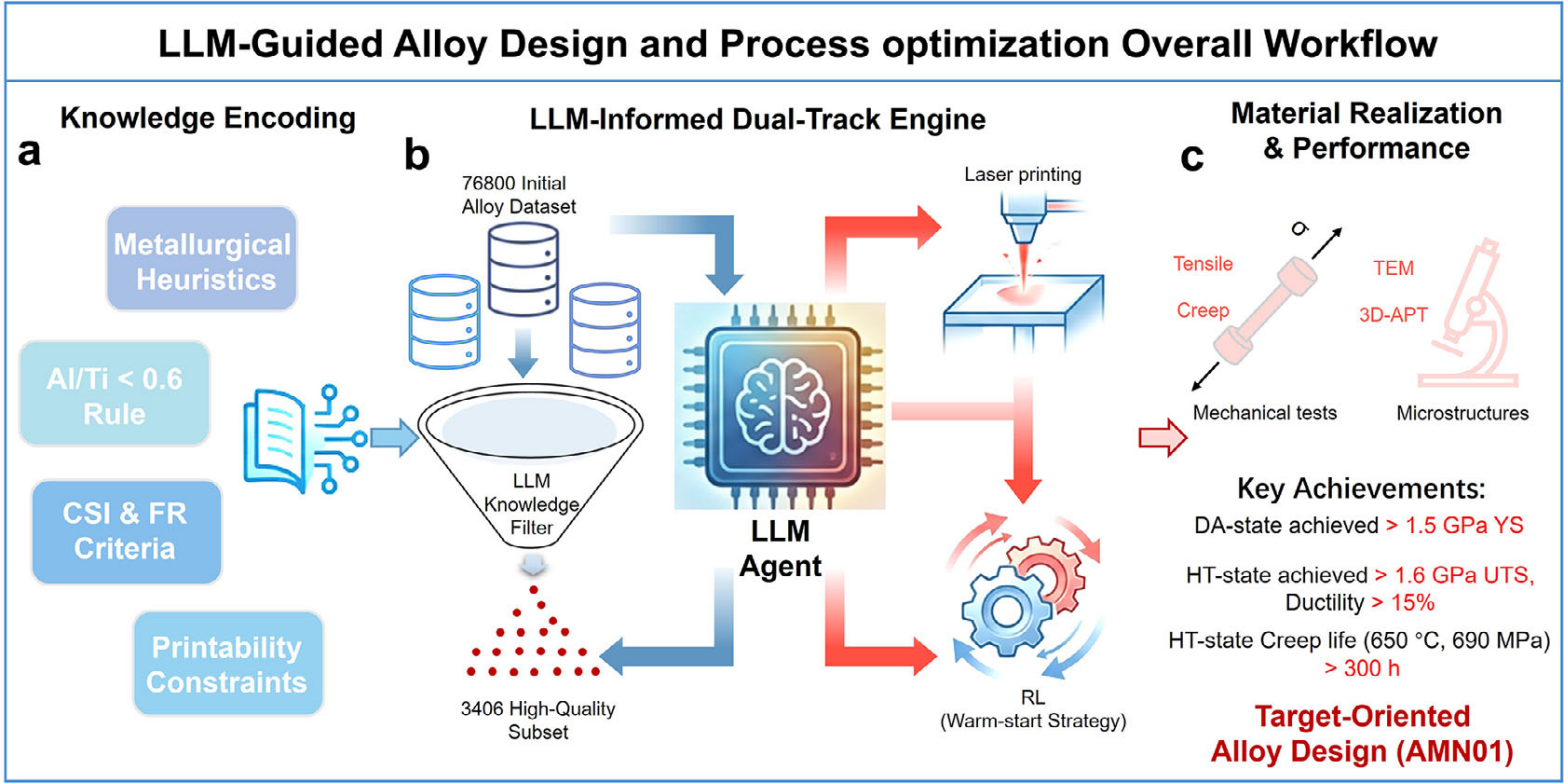
The overall process of knowledge‐augmented dual‐track AI framework. (a) Transformation of empirical heuristics (e.g., Al/Ti rules, crack susceptibility criteria) into structured prompts for the Large Language Model (LLM). (b) Rapid search space reduction of 76 800 candidates via LLM‐guided heuristic pruning. Injection of LLM‐distilled process priors into a Reinforcement Learning (RL) agent to enable safety‐constrained autonomous exploration. (c) Successful discovery and validation of the ultra‐high‐performance AMN01 superalloy.

In this work, we present a knowledge‐augmented dual‐track AI framework to accelerate the synergistic optimization of composition and additive manufacturing processes for next‐generation superalloys, as shown in Figure 1. By integrating LLM‐based heuristic pruning with informed reinforcement learning, we successfully navigated the vast design space to discover AMN01, a novel Ni‐based superalloy. Experimental validation confirms that AMN01 exhibits exceptional printability across a broad processing window without microcracking, delivering an ultimate tensile strength of 1607.9 MPa and an elongation of 15.1%, outperforming state‐of‐the‐art additively manufactured superalloys. Furthermore, its creep resistance at 650°C surpasses benchmark crack‐free alloys. Through multi‐scale microstructural analysis and atom probe tomography, we reveal the synergistic strengthening mechanisms involving nanoscale cellular dislocation networks, precisely partitioned γ' precipitates, and deformation‐induced twins. This study not only delivers high‐performance material but also establishes a generalizable paradigm that bridges symbolic physical laws with autonomous experimentation, paving the way for the intelligent development of extreme‐environmental materials.

## 2 Results

### 2.1 Knowledge‐Informed Alloy Design

**FIGURE 2:**
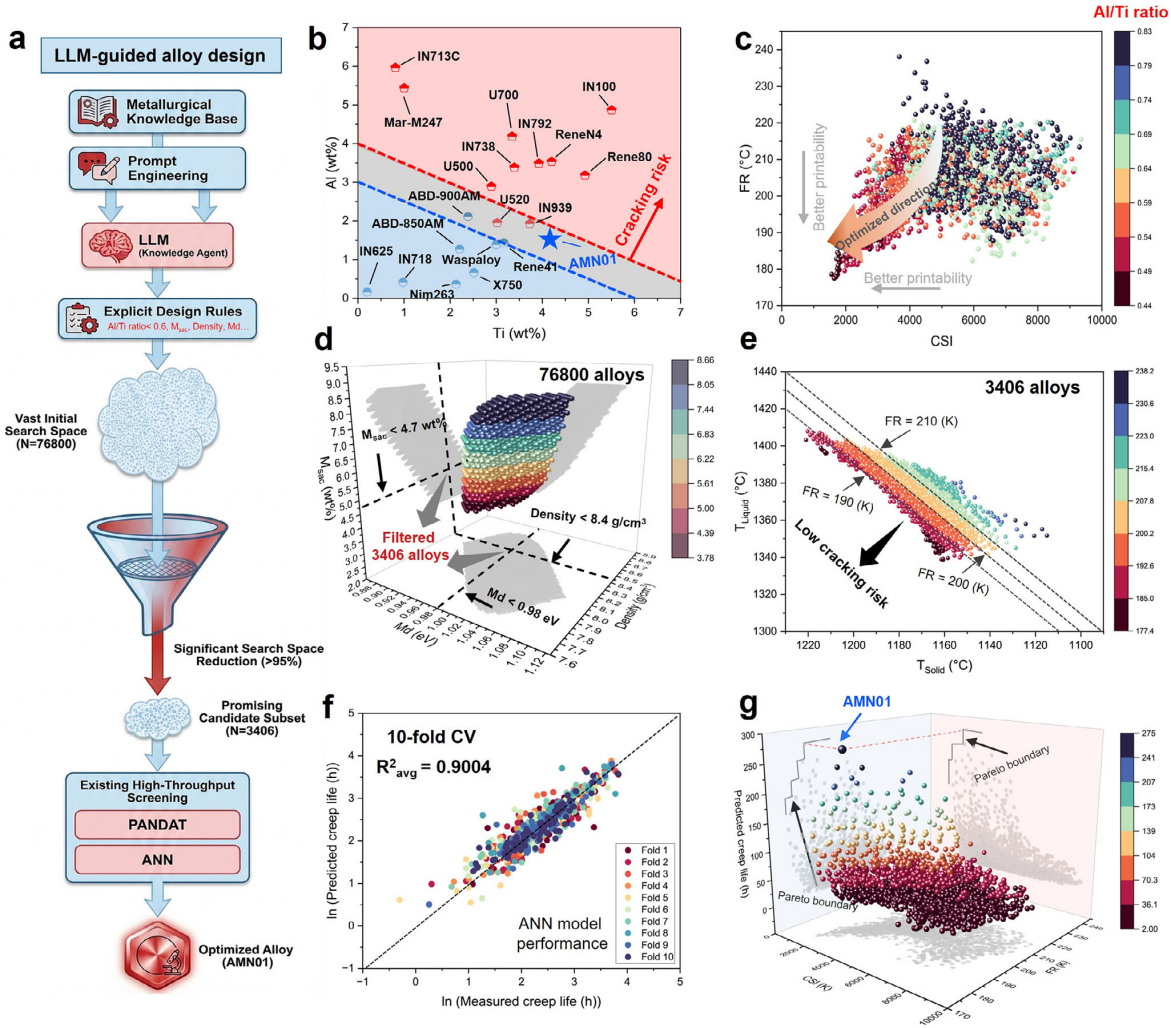
Knowledge‐informed compositional design and heuristic screening. (a) Workflow of LLM‐guided explicit rule generation. (b) Al and Ti content distribution in existing superalloys and their laser weldability boundary. (c) Relationship between Al/Ti ratios and calculated solidification parameters (FR and CSI). (d) The heuristic filtering process reducing the initial 76 800 candidates to a subset of 3406 alloys. (e) Solidification characteristics map (*T*_liquid_ vs *T*_solid_) of the promising subset. (f) 10‐fold cross‐validation performance of the ANN model for creep life prediction. (g) 3D Pareto optimization identifying AMN01 at the performance frontier.

To accelerate the discovery of crack‐free superalloys, we developed an LLM‐guided alloy design paradigm, as illustrated in Figure 2a. In this framework, a metallurgical knowledge base was integrated into an LLM knowledge agent via prompt engineering. The LLM performed heuristic reasoning to synthesize explicit design rules from unstructured expertise, bridging the gap between historical metallurgical laws and numerical optimization. A central insight operationalized by the LLM is the decisive role of the Al/Ti ratio in suppressing solidification cracking. As visualized in Figure 2b, an analysis of existing superalloys reveals that laser‐printable alloys (e.g., IN718, IN625) consistently maintain a lower Al+Ti concentration, whereas high‐strength cast alloys (e.g., IN713C, Mar‐M247) fall into the high‐risk cracking zone. To quantitatively refine this rule, thermodynamic calculations of the Freezing Range (FR) [17] and Crack Susceptibility Index (CSI) [18] were conducted (Figure 2c). It should be noted that due to LPBF's extremely high cooling rate (10^5^−10^6^ K/s), we employed the Scheil–Gulliver nonequilibrium solidification model when calculating FR and CSI in PANDAT. The results confirm the LLM's reasoning: a lower Al/Ti ratio effectively steers candidate alloys toward the optimized direction, characterized by significantly reduced FR and CSI values, thereby expanding the printability window.

Using these LLM‐distilled rules (Al/Ti ratio < 0.6, density < 8.4 g/cm^3^ (Figure S1), and Md < 0.98 eV (calculated by Table S1) [19], M_sac_ < 4.7 wt.% [20, 21] (Figure S2), we performed significant search space reduction on a vast initial library of 76 800 alloy compositions. Instead of direct semantic evaluation, the LLM was utilized as a reasoning agent to extract unstructured knowledge from literature and translate it into executable Python screening scripts, as shown in Table S3. This code‐driven heuristic pruning efficiently compressed the initial search space of 76 800 candidates by >95%, yielding a promising subset of 3406 candidate alloys, as shown in Figure 2d. Notably, relying solely on phase diagram calculations (CALPHAD) proves prohibitively costly and impractical for large‐scale search spaces. For example, evaluating 76 800 candidate components would require weeks of cluster computing. In contrast, heuristic screening based on large language models enhances initial screening efficiency by several orders of magnitude, compressing the candidate space in just minutes. The solidification characteristics of this subset, mapped in Figure 2e, demonstrate that most candidates were successfully restricted to a narrow FR window (190–210 K), drastically mitigating the risk of solidification cracking during LPBF. To mathematically and statistically demonstrate the compositional diversity of the 3406 retained alloys, we conducted a distribution analysis of the data. As shown in Figure S11, the pruned subset maintained extremely broad and reasonable distribution ranges for all key alloying elements. The critical elements for solid solution strengthening and precipitate formation still exhibited a wide design span: cobalt (15–23 wt.%), chromium (12–17 wt.%), titanium (3.0–4.5 wt.%), and tantalum (2.0–4.0 wt.%), with no evidence of aggregation toward a single component level. Simultaneously, to ensure high‐temperature serviceability, an artificial neural network (ANN) was employed to predict the creep rupture life (as shown in Figures S3 and S10). The model, trained on 920 experimental datasets, achieved a 10‐fold cross‐validation R^2^ of 0.9004, indicating excellent predictive robustness (Figure 2f). Finally, by integrating CSI, FR, and predicted creep life into a 3D Pareto optimization (Figure 2g), AMN01 was identified on the Pareto frontier, the composition is in Table 1. This optimized composition successfully synchronizes the LLM‐informed printability requirements with superior high‐temperature performance (650°C/690 MPa).

**TABLE 1:** The nominal and actual chemical compositions (wt.%) of the AMN01 alloy.

AMN01	Al	Ti	Co	Cr	Ta	W	Mo	C	B
Nominal	2	4.5	23	17	2	4	2	0.03	0.005
Actual	1.81	4.15	22.9	17	1.78	3.82	2.04	0.025	0.004

### 2.2 LLM‐Augmented Autonomous Process Optimization

**FIGURE 3:**
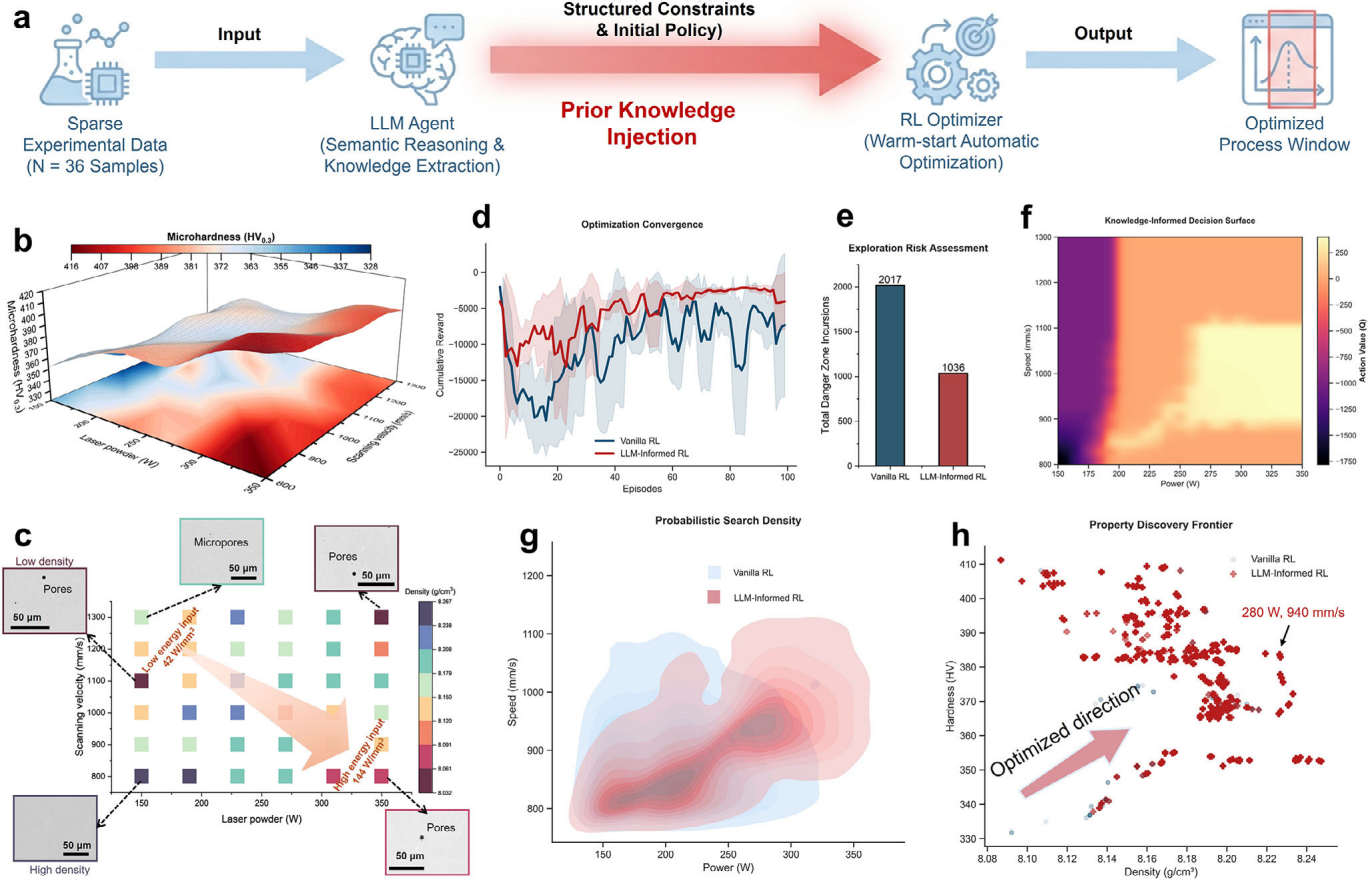
LLM‐informed Reinforcement Learning for autonomous process optimization. (a) Data‐to‐policy pipeline illustrating knowledge extraction and prior injection. (b, c) Experimental response surfaces for microhardness and density from 36 initial samples. (d) Convergence efficiency comparison between Vanilla RL and LLM‐informed RL. (e) Exploration risk assessment showing significantly reduced failure counts via LLM guidance. (f) Knowledge‐informed decision surface (Q‐table) displaying the agent's learned preference. (g) Probabilistic search density maps (KDE) highlighting the concentrated search trajectory. (h) Property discovery frontier pinpointing the optimal window for AMN01 (P = 280 W, V = 940 mm/s).

While the AMN01 alloy demonstrated robust printability (as shown in Figure S4), identifying the optimal synergistic combination of laser power (P) and scanning speed (V) to simultaneously maximize density and microhardness remains a high‐dimensional challenge [22, 23]. Traditional grid‐based experimental mapping (Figure 3b,c) provides essential snapshots of the process‐property landscape but is limited by data sparsity (N = 36). To overcome this, we developed an LLM‐informed Reinforcement Learning (RL) framework, as illustrated in Figure 3a. Unlike vanilla RL (Figure S5), which starts with zero knowledge [24], our approach utilizes an LLM agent to perform semantic reasoning and knowledge extraction from the sparse experimental data. This distilled expertise is then transformed into structured constraints and a prior policy, which is injected into the RL optimizer to enable a “warm‐start” autonomous search.

The performance enhancement brought by LLM integration is quantitatively validated through comparative training. As shown in the optimization convergence curves (Figure 3d), the LLM‐informed RL (red) achieved rapid and stable convergence within minimal episodes, whereas the Vanilla RL (blue) exhibited erratic exploration with significantly lower cumulative rewards. Crucially, the Exploration Risk Assessment (Figure 3e) reveals that the LLM‐informed agent effectively bypassed physically unviable regimes (e.g., the high‐risk cracking or lack‐of‐fusion zones at low power), reducing the total danger zone incursions by over 50%. The logic behind this efficiency lies in the Knowledge‐Informed Decision Surface (Figure 3f). The LLM‐injected prior knowledge reshaped the agent's brain (Q‐table), assigning high action values to high‐potential regions and heavy penalties to risk zones. This is further visualized in the Probabilistic Search Density map (Figure 3g), where the informed agent's search trajectory is precisely concentrated in the metallurgical “sweet spot”, while the vanilla agent scattered its efforts across low‐performance areas. Consequently, the LLM‐RL framework successfully identified the Property Discovery Frontier (Figure 3h), pinpointing the optimal processing window (P = 280 W, V = 940 mm/s) that delivers a superior balance of ultra‐high hardness and full densification, breaking through the Pareto boundary of the initial 36 samples.

### 2.3 Mechanical Properties

**FIGURE 4:**
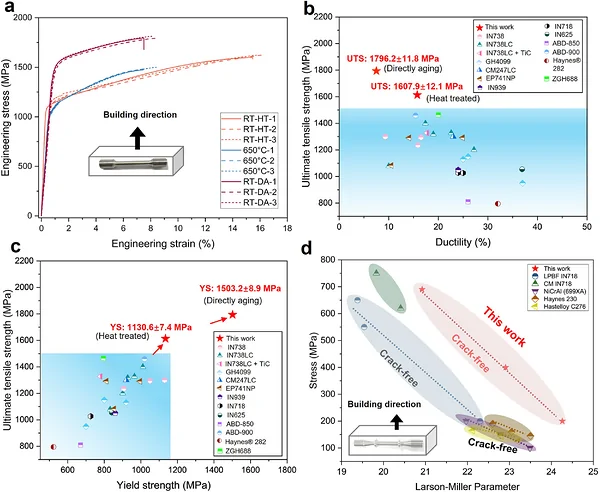
Mechanical properties and ASTM creep life test results of the alloy. (a) The engineering stress–strain curves of the AMN01 alloy at different test temperatures, demonstrating its high yield strength and tensile strength. (b) A comparison of tensile strength and elongation between the AMN01 alloy and other superalloys manufactured by LPBF at room temperature [20, 25, 26, 27, 28, 29, 30, 31, 32, 33]. (c) A comparison of tensile strength and yield strength between the AMN01 and other alloys under room temperature conditions. (d) The creep performance of the AMN01 alloy at different temperatures [34, 35, 36, 37], showing its excellent high‐temperature creep resistance compared to other crack‐free LPBF superalloys. Larson‐Miller parameter, *LMP* =  *T* (*C*+ log*t*_r_)  ×  10^−3^, *C* =  20.

The synergistic optimization of composition and LPBF processing via the LLM‐informed framework culminated in the exceptional mechanical response of the AMN01 alloy. To evaluate its service potential, specimens fabricated under the RL‐optimized window (P = 280 W, V = 940 mm/s) were subjected to direct aging (DA) and solution‐plus‐aging (HT) heat treatments, detailed schedules are provided in Figure S8. Due to the high heat treatment temperature, residual stresses can be effectively released; therefore, this paper does not conduct a further in‐depth analysis of residual stresses. As shown in Figure 4a, the RT‐DA condition delivers an ultra‐high yield strength (YS) of 1503.2 ± 8.9 MPa and an ultimate tensile strength (UTS) approaching 1.8 GPa, albeit with a moderate elongation of 8.1% ± 0.5%. Upon HT, the alloy achieves a superior strength‐ductility balance, maintaining an YS of 1130.6 ± 7.4 MPa and a UTS of 1607.9 ± 12.1 MPa, while the elongation significantly increases to 15.1 ± 0.9%. Notably, even at an elevated temperature of 650°C, the HT‐AMN01 alloy retains a remarkable YS of 1044 ± 9.2 MPa and a UTS near 1500 MPa, demonstrating robust high‐temperature structural stability. To highlight the competitive edge of AMN01, we benchmarked its RT performance against state‐of‐the‐art LPBF‐fabricated superalloys. In the UTS vs. Ductility map (Figure 4b), AMN01 occupies the topmost quadrant, significantly outperforming benchmark alloys such as IN718, IN738LC, and the ABD series. This superiority is further emphasized in the UTS vs. YS comparison (Figure 4c), where AMN01 defines a new performance boundary for crack‐free additively manufactured superalloys.

Furthermore, the high‐temperature creep resistance, a critical indicator for aerospace applications, was evaluated using the Larson‐Miller Parameter (LMP) vs. stress relationship (Figure 4d). Under various stress levels and temperatures, AMN01 exhibits creep lives that far exceed those of reported crack‐free LPBF superalloys (e.g., IN718, Haynes 230) and even surpasses the cast CM‐IN718. In addition, we evaluated the microstructural stability (Figure S13) and low‐cycle fatigue performance (Figure S14) of the alloy, further demonstrating its potential for widespread application. In summary, the ability of AMN01 to combine excellent creep resistance with high printability and zero‐crack characteristics highlights its immense potential for next‐generation hot‐section components, validating the effectiveness of our knowledge‐informed design strategy.

## 3 Discussion

**FIGURE 5:**
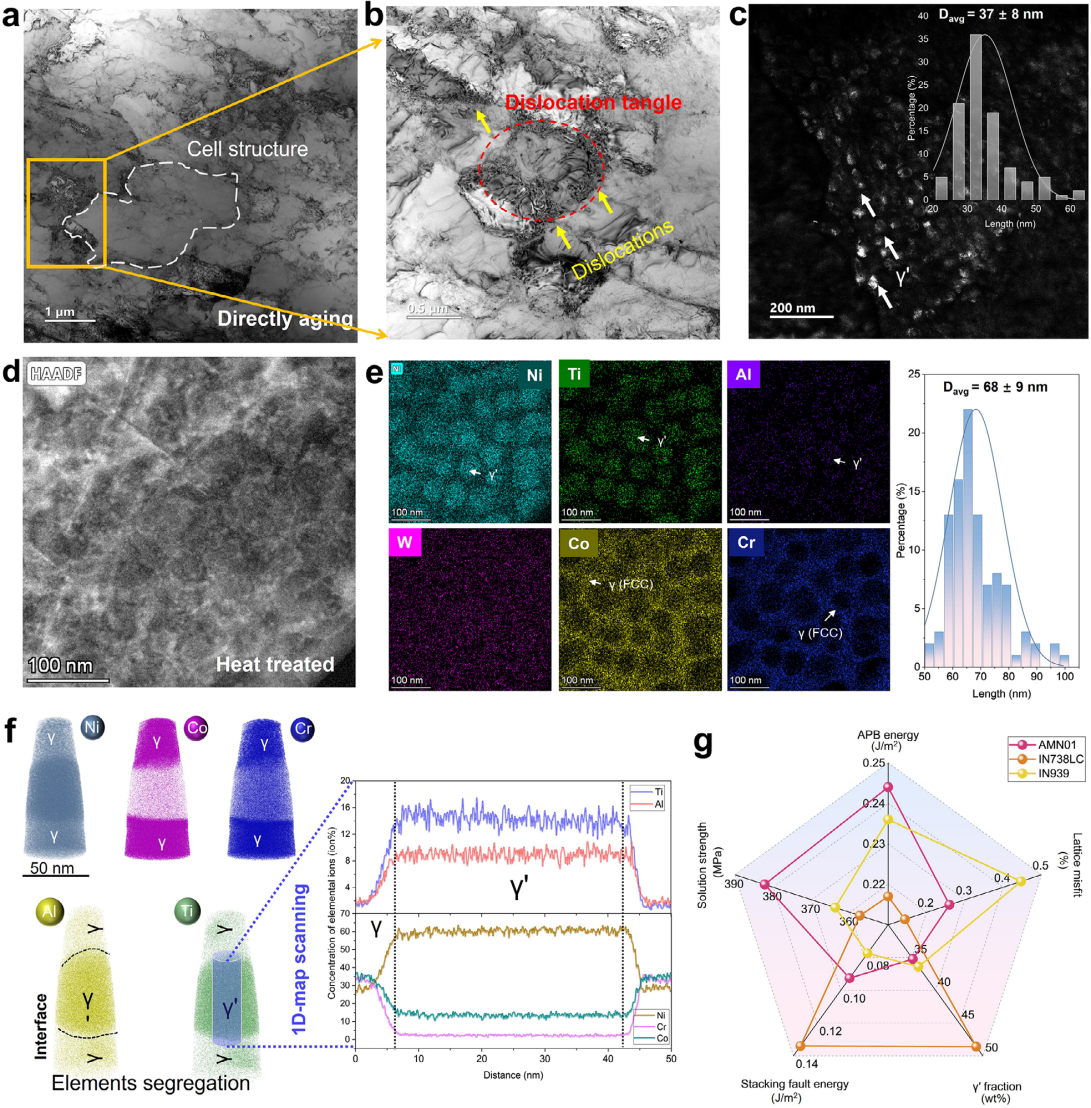
Multi‐scale microstructural characterization and thermodynamic genetic profile. (a, b) TEM images of the DA state revealing cellular substructures and dislocation tangles. (c) Size distribution of ultra‐fine γ' precipitates in the DA state. (d, e) HAADF‐STEM and EDX elemental maps of the HT state showing stable γ/γ' architecture. (f) 3D Atom Probe Tomography (APT) reconstructions and 1D concentration profiles confirming precise Ti/Al partitioning across the interface. (g) Multi‐dimensional radar chart benchmarking the theoretical physical properties (APB energy, SFE, and solution strength) of AMN01 against IN738LC and IN939.

The microstructure characteristics of AMN01 are comprehensively analyzed across multiple length scales in Figure 5. In the DA state, the alloy exhibits a hierarchical architecture featuring well‐defined cellular substructures (Figure 5a). Higher magnification reveals dense dislocation tangles concentrated along these cellular boundaries (Figure 5b), which are effectively stabilized by ultra‐fine, coherent γ' precipitates with an average diameter of 37 ± 8 nm (Figure 5c) [38]. This high density of obstacles to dislocation motion provides the microstructural basis for the alloy's ultra‐high yield strength (>1.5 GPa) [39, 40]. Following the HT state, the microstructure transforms into a stable, two‐phase γ/γ' structure, as shown in the HAADF‐STEM image (Figure 5d). The elemental distribution maps obtained via EDX (Figure 5e) confirm that the γ' precipitates coarsen to 68 ± 9 nm while maintaining a uniform spatial distribution. To further probe the atomic‐scale chemistry of these precipitates, 3D Atom Probe Tomography (APT) was employed (Figure 5f). The 3D reconstructions and corresponding 1D concentration profiles across the γ/γ' interface demonstrate a sharp partition of elements: Ti (13.6 at.%) and Al (9.2 at.%) are precisely enriched in the L1_2_ phase, while Cr and Co are rejected into the matrix [41].

This precise chemical partitioning is the physical realization of the LLM‐informed design goals. As summarized in the multi‐dimensional property comparison (Figure 5g), AMN01 was intentionally engineered to achieve a superior thermodynamic profile. The Ti‐rich nature of the γ' phase (Ti > Al) directly supports the predicted high anti‐phase boundary (APB) energy (∼0.244 J/m^2^) [42], which maximizes the energy barrier for dislocation shearing [43]. To further elucidate the creep mechanism of the AMN01 alloy, the creep fracture cross‐sections under 650°C/690 MPa were characterized by TEM (Figure S12). Microstructural analysis revealed no significant coarsening or cavity aggregation of the γ' phase, indicating excellent microstructural stability throughout the testing cycle. Microscopic deformation was primarily driven by continuous stacking faults (SFs) and deformation twins spanning the γ/γ' phases. Notably, numerous stacking faults penetrated the γ' precipitate phase, demonstrating that incomplete dislocation shear dominated the deformation mechanism. At the interfaces, high APB energy hindered direct cleavage by perfect dislocations, facilitating the entry of leading incomplete dislocations into the γ' phase and the formation of superlattice intrinsic stacking faults. The dense stacking fault network and twin interfaces effectively impeded subsequent dislocation slip, thereby conferring superior creep resistance to the alloy at the macroscopic level. Simultaneously, the solid‐solution strength is significantly elevated (∼390 MPa) compared to IN738LC and IN939. Crucially, the lower stacking fault energy (SFE) of AMN01 provides the thermodynamic prerequisite for activating stacking fault interactions [44], which underpins the alloy's exceptional ductility.

**FIGURE 6:**
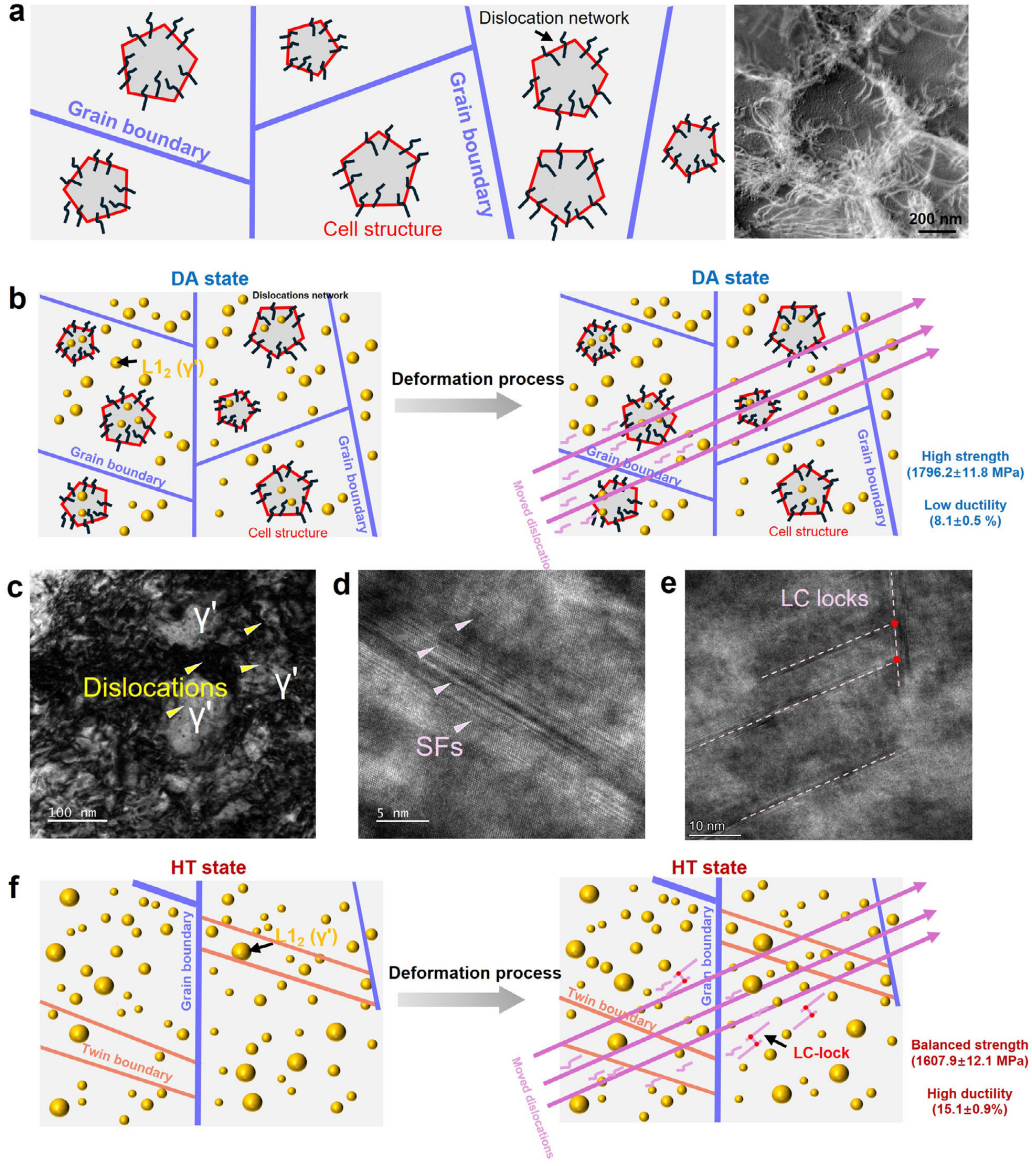
Deciphering the multi‐tier strengthening and plasticity‐enhancing mechanisms. (a, b) Schematic and STEM image illustrating the dislocation‐cell boundary pinning effect in the DA state. (c) Dislocation pileups at γ/γ' interfaces verifying the high shear resistance of Ti‐rich precipitates. (d, e) High‐resolution TEM capturing stacking faults (SFs) and immobile LC locks. (f) Comprehensive deformation model highlighting the synergy of annealing twins, γ' precipitates, and LC‐lock‐induced strain hardening in the HT state.

The fundamental origin of the superior strength‐ductility synergy in AMN01 is deciphered through the deformation mechanisms illustrated in Figure 6. In the as‐built and DA states, the alloy inherits a unique hierarchical architecture from the LPBF process, characterized by nanoscale cellular dislocation networks (Figure 6a). As shown in the schematic of Figure 6b, these cell boundaries, enriched with heavy dislocation tangles, act as potent barriers to dislocation glide. This high density of cellular walls provides a strong Hall‐Petch‐like strengthening effect, which, combined with the coherent γ' precipitates, pushes the yield strength to the 1.5 GPa regime. However, this restrictive network also limits dislocation mean free path, leading to premature strain localization and limited ductility.

The transition to high ductility in the HT state is driven by a profound microstructural reorganization facilitated by the LLM‐optimized chemistry. During solution treatment, the LPBF‐induced cellular substructures are erased by static recrystallization, accompanied by the formation of abundant annealing twins (Figure 6f). These twin boundaries effectively subdivide the grains and provide additional obstacles to slip without the deleterious effect of high residual stresses. As visualized in Figure 6c, dislocations predominantly pile up at the γ/γ' interfaces, directly confirming the high shear resistance (high APB energy) of the Ti‐rich L1_2_ precipitates.

Furthermore, the low stacking fault energy (SFE) of AMN01, a key feature of LLM design, activates diverse plasticity‐enhancing events. High‐resolution TEM captures the formation of extensive stacking faults (SFs) (Figure 6d) and the development of Lomer–Cottrell (LC) locks [45, 46, 47] (Figure 6e). These immobile LC‐locks, formed by the intersection of Shockley partials on different slip planes [48], serve as strong forest dislocation barriers that promote sustained work hardening [49]. In essence, the HT state achieves a balanced mechanical response by replacing the rigid cellular network with a synergy of annealing twin boundaries (as shown in Figures S6 and S7), high‐APB shear resistance, and LC‐lock‐induced strain hardening.

## 4 Conclusion

In summary, we have developed a knowledge‐augmented dual‐track AI framework that transcends the limitations of traditional “black‐box” data‐driven models by integrating Large Language Models (LLMs) as reasoning agents. By operationalizing metallurgical heuristics, this framework achieved a >95% search space reduction and enabled a “low‐failure” process optimization for the successful discovery of AMN01, a crack‐free superalloy with ultra‐high ultimate tensile strength (1607.9 ± 12.1 MPa) and exceptional ductility (15.1 ± 0.9%). Multi‐scale characterization confirmed that the LLM‐informed design successfully prioritized high‐APB‐energy precipitates and low‐SFE matrix interactions, establishing a multi‐tier strengthening architecture involving nanoscale L1_2_ precipitates, cellular networks, and deformation‐induced LC locks. This study establishes a generalizable, knowledge‐informed paradigm for the accelerated development of high‐performance materials in data‐scarce and high‐cost engineering environments.

## 5 Methods

### 5.1 Alloy Design Process

A knowledge‐informed alloy design paradigm was developed by integrating a Large Language Model (LLM, Qwen3 & Gemini 3‐pro) with high‐throughput thermodynamic calculations. Initially, a metallurgical knowledge base containing empirical heuristics for superalloy weldability, microstructure stability, and phase equilibrium was encoded into the LLM through structured prompt engineering, as shown in Table S3. The LLM acted as a reasoning agent to distill unstructured expertise into explicit design rules. The properties and performance calculations involved in this study cover a range of indicators, including density, microstructural stability, γ′ fraction, strain‐age cracking index, FR (Freezing Range), CSI (Crack Susceptibility Index), and creep rupture. The calculation processes involve thermodynamic calculations and machine learning models. The specific methods and procedures are detailed in Supporting File S1.

### 5.2 Preparation of Alloy Powders and LPBF Printing Process

In this study, the alloy powders suitable for Laser Powder Bed Fusion (LPBF) were prepared using argon gas atomization. Metal ingots with a purity of 99.9% were selected and weighed according to the nominal alloy composition, then fed into the argon gas atomization system. The metal ingots were atomized into powder particles through the gas atomization process. Subsequently, powders with a particle size *D*_avg =_ of 34.8 ± 5.7 µm were sieved out, as shown in Figure S9. This particle size range is highly compatible with the characteristics of the LPBF process, ensuring good flowability of the powders during laser melting.

The printing process was carried out using the Hans Laser M100 equipment, with stainless steel 304 used as the substrate material. After process optimization, the laser power was set to 280 W, the scanning speed to 940 mm/s, the hatch spacing to 0.09 mm, and the layer thickness to 0.03 mm. During each layer of printing, a scanning strategy with a 67° rotation between layers was employed, and argon gas was used as the protective gas throughout the process.

### 5.3 Process Parameter Optimization Based on Reinforcement Learning

The LPBF process parameters (laser power, P; scanning speed, V) were optimized using an LLM‐augmented Reinforcement Learning (RL) framework. To overcome the “cold start” challenge in traditional RL, the LLM was employed to perform semantic reasoning on 36 preliminary experimental snapshots.

By identifying underlying process‐property trends, the LLM synthesized a prior knowledge policy, which was mathematically encoded into the initial Q‐table of the Q‐learning algorithm. In this study, the Q‐learning reinforcement learning algorithm was employed to optimize the LPBF process, focusing on the parameters of scanning speed and laser power. The construction methods of key steps in reinforcement learning, such as the agent's action space and environmental feedback, are detailed in Supporting File S1. To mathematically implement the warm start strategy, the semantic reasoning distilled by the LLM was translated into explicit constraints within the initial Q‐table, *Q*_0_(*s*,*a*), as shown in Table S4. The state space is defined by Laser Power (P) and Scanning Speed (V). The LLM‐informed prior policy initialized the action‐values using the following mathematical formulation:

$$Q_{0}((P,V),a)=\left\{\begin{array}{cc} -1000 & ifP\leq 190\;W\\ +400 & ifP\geq 270\;Wand\;900\leq V\leq 1100\;mm/s\\ 0 & otherwise \end{array}\right.$$

This non‐uniform matrix acts as a hard mathematical constraint. It inherently penalizes the RL agent for exploring physically unviable regimes while encouraging exploitation in the high‐potential optimal window verified by the LLM. Here, the danger zone or high‐risk region was quantitatively defined as the parameter space where the laser power P <190 W. Within this threshold, the volumetric energy density is critically insufficient, unavoidably leading to severe lack‐of‐fusion macro‐defects and drastic mechanical degradation.

The density and microhardness of the samples obtained under different process conditions were measured using an electronic balance with a precision of 0.0001 g and a 320HVS‐5 digital display Vickers hardness tester.

### 5.4 Microstructure Characterization

The Tescan‐Mira4 scanning electron microscope was used to observe the microstructure of the alloy. The EBSD probe, NordlysMax2, was employed to characterize the crystal structure, with an operating voltage of 20 kV, a current of 10 nA, and a scan step size of 1.0 µm. The TESCAN‐AMBER focused ion beam scanning electron microscope was utilized to prepare transmission samples. The double aberration‐corrected Spectra‐300 TEM was used to capture high‐resolution atomic images and perform EDX analysis of the samples, with an operating voltage of 300 kV. The average size and distribution of γ' precipitates were statistically quantified from at least five representative TEM images using ImageJ software, measuring a minimum of 200 particles per condition to ensure statistical reliability. The Helios 5UC FIB‐SEM was employed to prepare 3D atom probe samples, and the LEAP 5000XR laser imaging mode was used to conduct near‐atomic‐resolution 3D compositional analysis.

### 5.5 Mechanical Performance Test

The tensile properties were tested in accordance with GB/T228.2‐2015, using a 68FM‐100 universal electronic testing machine. The total length of the tensile test sample is 71 mm, with a gauge length of 25 mm and a diameter of 5 mm. The testing environmental conditions were 23°C, 650°C. The tensile strain rates were 0.015 mm/min before yielding and 12 mm/min after yielding at room temperature. The high‐temperature strain rates were 7 × 10^−5^ mm/s before yielding and 2.5 mm/min after yielding. The high‐temperature creep properties were tested in accordance with ASTM standards, using an RWS100 electronic creep testing machine. The total length of the creep test sample is 77 mm, with a gauge length of 26 mm and a diameter of 5 mm. The testing conditions were 650°C‐690 MPa, 800°C‐400 MPa, and 900°C‐200 MPa.

## Author Contributions

J.Y. designed the computational and experimental strategy, built the high‐throughput thermodynamic and machine‐learning workflows, and wrote the manuscript. J.W. conducted microstructural characterization, collected density, hardness and microscopy data, and revised the manuscript. J.S. and F.W synthesized the alloy powder, carried out all LPBF builds and post‐processing, and analyzed the mechanical data. Z.W. and L.W. performed TEM, HRTEM, EDX and atom‐probe tomography analyses, interpreted the microstructural results, and revised the manuscript. F.L. and Y.L. guided the machine‐learning modeling, interpreted creep‐life predictions, and reviewed the manuscript. L.T. and L.H. supervised the overall project, acquired funding, coordinated resources, and critically revised the manuscript.

## Conflicts of Interest

The authors declare no conflicts of interest.

## Supporting information




**Supporting File**: advs76036‐sup‐0001‐SuppMat.docx.

## Data Availability

The data that support the findings of this study are available from the corresponding author upon reasonable request.
