# Supplementary material for: Large Language Model‐Informed Dual‐Track AI Framework for the Synergistic Design of Crack‐Free and High‐Strength Superalloys
Source: Adv Sci (Weinh). 2026 Jun 9:e76036. Online ahead of print. doi: 10.1002/advs.76036 (PMC13336658; doi:10.1002/advs.76036)
Supplement: Supplementary file 1 — Supporting File: advs76036‐sup‐0001‐SuppMat.docx. [file ADVS-9999-e76036-s001.docx]

**Supplementary file 1**

**Density calculation**

In this study, the density of nickel-based superalloys was calculated using a modified version of the Caron model. The performance of the modified model is compared with that of the original Caron model as shown in Fig. 1. It can be observed that, at lower density ranges, for example, for DD3, SC16, and CMSX-6 alloys, the Caron density model tends to underestimate the alloy density. The updated density model exhibits excellent predictive capability over a broader range of densities.


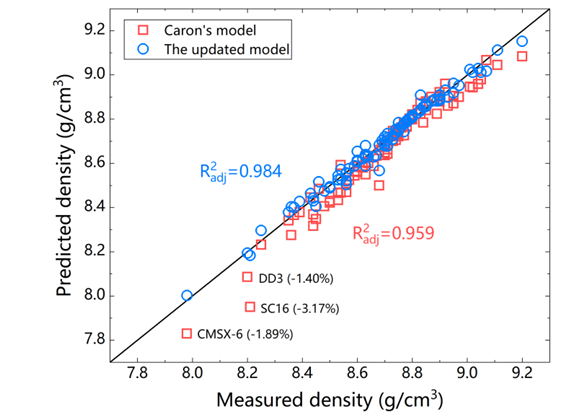


**Fig. S1 Performance of the updated density prediction model.**

The updated density modeling formula is as follows:

$$\begin{aligned} \text{ρ}\text{(g/c}\text{m}^{\text{3}}\text{)\&=8.939−0.0014}\text{c}_{\text{Co}}\text{−0.0197}\text{c}_{\text{Cr}}\text{+0.0125}\text{c}_{\text{Mo}} \\ \text{\&+0.0436}\text{c}_{\text{W}}\text{+0.0508}\text{c}_{\text{Re}}\text{+0.0194}\text{c}_{\text{Ru}} \\ \text{\&−0.1281}\text{c}_{\text{Al}}\text{−0.0498}\text{c}_{\text{Ti}}\text{+0.0409}\text{c}_{\text{Ta}} \end{aligned}$$

where *c_i_* denotes the mass percentage of alloying element *i*.

The theoretical density of AMN01, calculated based on its alloy composition, is 8.373 g/cm^3^. The maximum density measured experimentally using the drainage method for the additive manufacturing samples was 8.267 g/cm^3^, indicating a numerical discrepancy between theoretical calculations and experimental measurements. However, this deviation is physically reasonable and entirely consistent with expectations. The empirical Caron formula calculates the maximum theoretical density under the assumption of an absolutely ideal, 100% dense, and defect-free crystallographic state. In contrast, components fabricated via laser selective melting (LPBF) inevitably contain trace inherent metallurgical microdefects (such as residual pores or shrinkage cavities) and may exhibit slight elemental volatilization due to intense laser-melt pool interactions. Thus, this anticipated discrepancy does not undermine the model's validity but rather reflects the natural physical gap between ideal composition calculations and actual additive manufacturing processes.

**Microstructure stability**

Superalloys are materials that can operate stably at high temperatures over extended periods and are widely used in high-temperature components such as aeroengines and gas turbines. Therefore, their microstructural stability needs to be carefully assessed. In this study, the New PHACOMP method was employed to predict the microstructural stability of superalloys. The specific calculation method is as follows:

$$\overset{\text{¯}}{\text{Md}}\text{=}\sum_{\text{i}}^{\text{n}} \text{ }\text{x}_{\text{i}}\text{M}\text{d}_{\text{i}}$$

In the formula, *x_i_* represents the atomic fraction of alloying element *i*, while *Md_i_* represents the energy level of the d-orbitals for element *i*. The *Md* values for common elements in nickel-based superalloys are summarized in Table A1.

| **Table S1** d-orbital energy level (eV) of element in superalloys | | | | | | | | | | |
| --- | --- | --- | --- | --- | --- | --- | --- | --- | --- | --- |
| Element | Cr | Co | Mo | W | Al | Ti | Ta | Re | Ni |  |
| *Md* | 1.142 | 0.771 | 1.55 | 1.655 | 1.9 | 2.271 | 2.224 | 1.267 | 0.717 |  |

**Strain age cracking index**

Strain-age cracking (SAC) in nickel-based alloys typically occurs during thermal cycling or subsequent heat treatment processes, resulting from the superposition of residual stresses and additional stresses caused by the precipitation of the γ′ phase from the solid solution. To quantify the susceptibility to strain-age cracking, Thompson proposed an empirical index, which was later modified by Reed. The index is based on the assumption that the formation of the γ′ phase reduces local ductility through the effective total fraction of γ′-forming elements (in weight percent), which include aluminum (Al), titanium (Ti), niobium (Nb), and tantalum (Ta). In this study, this index is used to measure the risk of strain-age cracking, and it is expressed as follows:

$$\text{M}_{\text{sac}}\text{=}\text{W}_{\text{Al}}\text{+0.5}\text{W}_{\text{Ti}}\text{+0.3}\text{W}_{\text{Nb}}\text{+0.15}\text{W}_{\text{Ta}}$$

The *W_x_* is the mass fraction of alloying element *X*.

**Freezing range**

To quantify the susceptibility to hot cracking, the focus is on the formation mechanisms of hot cracking during the solidification process. Initially, a model was proposed in the casting literature that used the difference between the liquidus temperature (*T_Liquidus_*) and the solidus temperature (*T_Solidus_*), i.e., the solidification range (FR), as a key parameter for assessing the cracking tendency. The liquidus and solidus temperatures were calculated using the solidification module of PANDAT_2022 software. Subsequently, this model was further applied to the nickel-based alloy system, as shown below:

$$\text{FR}\text{=}\text{T}_{\text{Liquidus}}\text{−}\text{T}_{\text{Solidus}}$$

Here, the solidus temperature is defined as the temperature when the solid fraction reaches a molar fraction of 0.99. Alloy compositions that result in a wide solidification range and the formation of low-melting-point solidification products (such as carbides, borides, and eutectics of γ/γ′ phases) increase the sensitivity to cracking.

**CSI index**

In this study, the criterion for controlling liquid-phase cracking during solidification is based on the Solidification Crack Susceptibility Index (CSI) proposed by Kou et al. Kou simplified the solidification process by assuming that two columnar dendrites grow axially side by side in the mushy zone. The lateral separation of dendrites induces tensile stress, which leads to crack formation, while the lateral growth of dendrites causes the two grains to bond together, resisting crack formation. Therefore, the flow of liquid along the grain boundary needs to be considered to suppress crack formation. The CSI criterion is thus defined as the steepness of the solid fraction versus temperature curve during the final stages of solidification, which can be calculated using the solidification module in PANDAT_2022 software. The formula is as follows:

$$\text{Kou}\text{=}\left| \frac{\text{dT}}{\text{d}\text{(}\text{f}_{\text{s}}^{\text{1/2}}\text{)}} \right|_{\text{f}_{\text{s}}^{\text{1/2}}\text{→1}}$$

The *f_s_* is the solid fraction. The CSI index is obtained near $\text{f}_{\text{s}}^{\text{1/2}}\text{=1}$, where the solid fraction is defined in the range of 0.87 to 0.94.

**γ′ conten and Msact**

Using the point calculation function in the phase field module of PANDAT_2022, the phase composition of alloys at specific temperatures can be obtained. Calculations of γ′ phase content at 900 °C were carried out. As shown in Fig. S2, *M_sac_* values increase with higher γ′ phase content. While increased γ′ phase content boosts mechanical properties, it reduces laser printability.


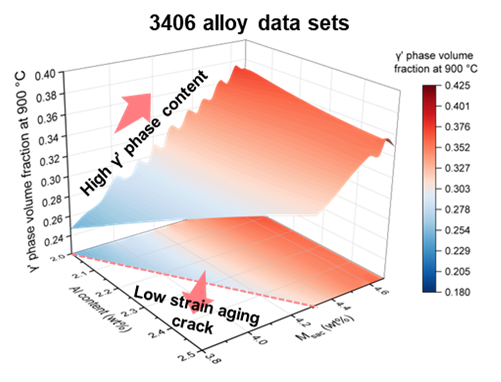


**Fig. S2 Thermodynamic calculations of the γ′-phase content.**

**Creep life prediction**

To predict the effect of alloy composition on creep rupture life, this study employed an Artificial Neural Network (ANN) combined with 15 new thermodynamic descriptors. The model was trained using an extensive dataset comprising 920 data points and optimized through ten-fold cross-validation, demonstrating excellent predictive performance with a mean validation result of 0.9004. The newly established compositional sample library was then used to predict creep life using this model. The structure of the neural network model is shown in Fig. S3.


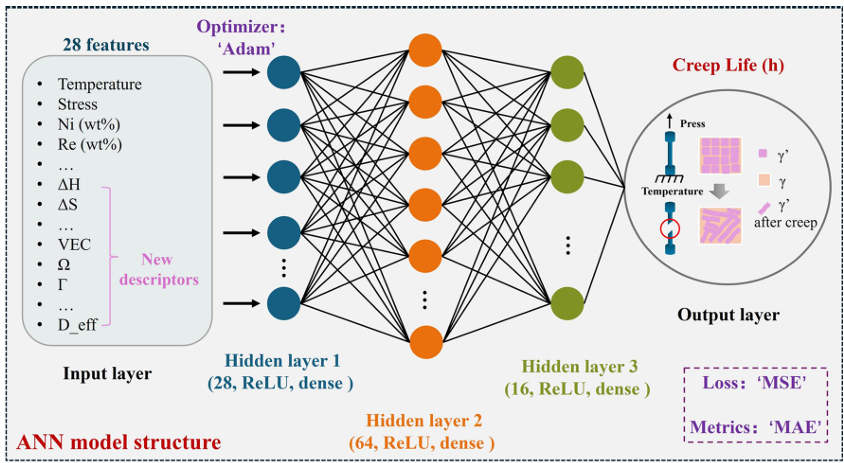


**Fig. S3 Structure of the ANN creep life prediction model.**

The thermodynamic parameters involved are shown in Table 2.

**Table S2 Thermodynamic parameters**

| **No.** | **Symbol & Formula** | **Meaning & Parameter** | **Ref.** |
| --- | --- | --- | --- |
| 1 | $\text{Δ}\text{S}_{\text{conf}}\text{=−}\text{R}\sum_{\text{i}\text{=1}}^{\text{n}} \text{ }\text{c}_{\text{i}}\text{ln⁡}\text{c}_{\text{i}}$  ($\Delta S_conf$) | Structural entropy. $\text{R}$, gas constant, 8.314 J/(mol·K); $\text{c}_{\text{i}}$, mole fraction of the *i*th element | [1] |
| 2 | $\text{Δ}\text{H}_{\text{mix}}\text{=}\sum_{\text{i}\text{=1,}\text{i}\text{≠}\text{j}}^{\text{n}} \text{ }\text{Ω}_{\text{ij}}\text{c}_{\text{i}}\text{c}_{\text{j}}$  ($\Delta H_mix$) | $\begin{aligned} \text{\&Mixing enthalpy of a solid solution }\text{Ω}_{\text{ij}}\text{(=4Δ}\text{H}_{\text{AB}}^{\text{mix}}\text{),regular} \\ \text{\&melt-interaction parameter between ith and jth elements;} \\ \text{\&Δ}\text{H}_{\text{AB}}^{\text{mix}}\text{,mixing enthalpy of binary liquid alloy.} \end{aligned}$ | [2] |
| 3 | $\text{δ}\text{=100×}\sqrt{\sum_{\text{i}\text{=1}}^{\text{n}} \text{ }\text{c}_{\text{i}}\left( \text{1−}\frac{\text{r}_{\text{i}}}{\bar{\text{r}}} \right)^{\text{2}}}$  ($\delta$) | Atomic size difference in multi-component alloys  $\bar{\text{r}}\left( \text{=}\sum_{\text{i}\text{=1}}^{\text{n}} \text{ }\text{c}_{\text{i}}\text{r}_{\text{i}} \right)$, average atomic radius; $\text{r}_{\text{i}}$, atomic radius of the *i*th element.‘ | [3, 4] |
| 4 | $\text{Δ}\text{χ}\text{=}\sqrt{\sum_{\text{i}\text{=1}}^{\text{n}} \text{ }\text{c}_{\text{i}}\text{(}\text{χ}_{\text{i}}\text{−}\bar{\text{χ}}\text{)}^{\text{2}}}$  ($\Delta \chi$) | Electronegativity difference between two alloy components $\bar{\text{χ}}\left( \text{=}\sum_{\text{i}\text{=1}}^{\text{N}} \text{ }\text{c}_{\text{i}}\text{χ}_{\text{i}} \right)$, average electronegativity; $\text{χ}_{\text{i}}$, Pauling  electronegativity for *i*th component. | [5] |
| 5 | $\text{VEC}\text{=}\sum_{\text{i}\text{=1}}^{\text{n}} \text{ }\text{c}_{\text{i}}\text{(}\text{VE}\text{C}_{\text{i}}\text{)}$  (VEC) | Valence electron concentration $\text{VE}\text{C}_{\text{i}}$, valence electron  concentration of *i*th. | [6] |
| 6 | $\text{T}_{\text{m}}\text{=}\sum_{\text{i}\text{=1}}^{\text{n}} \text{ }\text{c}_{\text{i}}\text{(}\text{T}_{\text{m}}\text{)}_{\text{i}}$  (Tm) | Melting point of n-elements alloy, rule of mixtures $\text{(}\text{T}_{\text{m}}\text{)}_{\text{i}}$, melting point of *i*th. | [7] |
| 7 | $\text{Ω=}\frac{\text{T}_{\text{m}}\text{Δ}\text{S}_{\text{conf}}}{\text{\vertΔ}\text{H}_{\text{mix}}\text{\vert}}$  ($\Omega$) | The ratio of the entropy changes *ΔS_con_*_f_ due to mixing to the heat change *ΔH_mix_* during mixing at the phase transition temperature *T_m_* is described. It can provide information about the thermodynamic properties of the mixing process—predicting the solid-solution formation. | [7] |
| 8 | $\text{Λ=}\frac{\text{Δ}\text{S}_{\text{conf}}}{\text{δ}^{\text{2}}}$  ($\Lambda$) | *ΔS_conf_* represents the entropy change of the system due to compositional changes, *δ* shows the difference in atomic size in the alloy, Λ can be understood as the ratio of the entropy change of the system to the square of the degree of lattice distortion, providing a measure of the sensitivity of the system to compositional changes. | [8] |
| 9 | $\text{γ}\text{=}\left[ \text{1−}\sqrt{\frac{\text{[(}\text{r}_{\text{s}}\text{+}\bar{\text{r}}\text{)}^{\text{2}}\text{−}{\bar{\text{r}}}^{\text{2}}\text{]/(}\text{r}_{\text{s}}\text{+}\bar{\text{r}}\text{)}^{\text{2}}}{\text{1−}\sqrt{\text{[(}\text{r}_{\text{L}}\text{+}\bar{\text{r}}\text{)}^{\text{2}}\text{−}{\bar{\text{r}}}^{\text{2}}\text{]/(}\text{r}_{\text{L}}\text{+}\bar{\text{r}}\text{)}^{\text{2}}}}} \right]$  ($\gamma$) | The ratio between the solid angles of the smallest and largest atoms $\bar{\text{r}}\left( \text{=}\sum_{\text{i}\text{=1}}^{\text{n}} \text{ }\text{c}_{\text{i}}\text{r}_{\text{i}} \right)$, average atomic radius; $\text{r}_{\text{L}}$ and $\text{r}_{\text{s}}$ are the radius of the largest and smallest atoms. | [9] |
| 10 | $\text{S}_{\text{E}}\text{=}\text{S}_{\text{E}}\text{(}\text{r}_{\text{i}}\text{,}\text{c}_{\text{i}}\text{,}\text{ξ}\text{)}$  (S_E) | *S_E_* denotes the excessive entropy of mixing, which describes the additional entropy change in an alloy system due to the mixing of different elements. It is related to factors such as the composition of the alloy, the atomic radius, and ξ, the atomic packing fraction of the n-element alloy range from 0.68 (BCC). | [10] |
| 11 | $\text{ϕ=}\frac{\text{S}_{\text{c}}\text{−}\text{S}_{\text{H}}}{\text{\vert}\text{S}_{\text{E}}\text{\vert}}\text{=}\frac{\text{S}_{\text{c}}\text{−Δ}\text{H}_{\text{mix}}\text{/}\text{T}_{\text{m}}}{\text{\vert}\text{S}_{\text{E}}\text{\vert}}$  ($\Phi$) | A single dimensionless thermodynamic parameter $\text{S}_{\text{c}}$, the configurational entropy of mixing for an ideal gas. $\text{ϕ}$: a dimensionless thermodynamic parameter that measures the non-ideality of a mixture by comparing the ratio of the ideal entropy of mixing to the actual entropy of mixing. | [10] |
| 12 | $\overset{\text{\_}}{\text{Δ}\text{V}\text{/}\text{V}}\text{=}\frac{\sum_{\text{i}\text{,}\text{j}} \text{ }\text{c}_{\text{i}}\text{c}_{\text{j}}\text{\vert}\text{V}_{\text{i}}\text{−}\text{V}_{\text{j}}\text{\vert}}{\text{(}\text{V}_{\text{i}}\text{+}\text{V}_{\text{j}}\text{)/2}}$  ($\overline {\Delta V/V} $) | The parameters of a novel two-dimensional structure map, $\text{V}_{\text{i}}\text{=}\frac{\text{4}}{\text{3}}\text{π}\text{r}_{\text{i}}^{\text{3}}$, atomic percentage of the *i*th component. *c_i_*: molar fraction of the ith component. $\overset{\text{\_}}{\text{Δ}\text{V}\text{/}\text{V}}$ often used to describe volume changes in an alloy or solution due to mixing of different components. | [11] |
| 13 | $\text{T}_{\text{γ}^{\text{'}}}\text{=1299.315−2.415}\text{w}_{\text{Co}}\text{−6.362}\text{w}_{\text{Cr}}\text{−2.224}\text{w}_{\text{Mo}}\text{+3.987}\text{w}_{\text{W}}\text{+0.958}\text{w}_{\text{Rc}}\text{+2.424}\text{w}_{\text{Ru}}\text{−2.603}\text{w}_{\text{Al}}\text{−4.943}\text{w}_{\text{Ti}}\text{+3.624}\text{w}_{\text{Ta}}$  (T_gamma_prime) | $\text{γ}^{\text{'}}$ phase dissolution temperature, the equation obtained from the multiple linear regression method. | [12] |
| 14 | $\text{δ}\text{=2(}\text{a}_{\text{γ}^{\text{'}}}\text{−}\text{a}_{\text{γ}}\text{)/(}\text{a}_{\text{γ}^{\text{'}}}\text{+}\text{a}_{\text{γ}}\text{)}$  (Misfit) | Lattice mismatch between $\text{γ}$ phase and $\text{γ}^{\text{'}}$ phase in superalloys. | [13] |
| 15 | $\text{D}_{\text{eff}}\text{=}\text{D}_{\text{0}}^{\text{eff}}\text{exp(−}\frac{\text{Q}_{\text{eff}}}{\text{RT}}\text{)}$  (D_eff) | The diffusion coefficients of the alloying elements in the $\text{γ}$ matrix phase are weighted and summed up as the effective diffusion coefficient of the superalloys. | [14] |

**Process Parameter Optimization and Q-learning**

To optimize the laser power (P) and scanning speed (V) in additive manufacturing, we employed a reinforcement learning algorithm based on Q-learning. The objective of optimization was to adjust these process parameters to achieve the desired density and hardness. Specifically, the goal was to use the Q-learning algorithm to identify an optimal combination of laser power (P) and scanning speed (V) that would meet the target standards for predicted density and hardness.

Fig. S4 shows the printed samples made under 6×6 process parameters. Hardness and density tests were done on these samples. The data collected served as the basis for reinforcement learning.


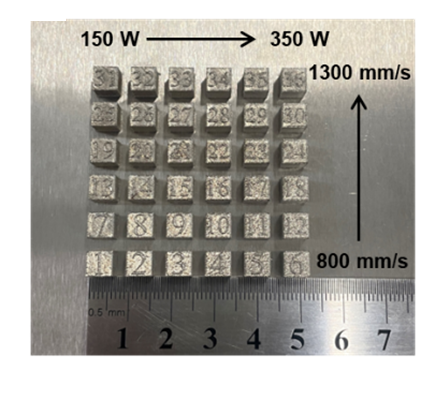


**Fig. S4 The CSU-AM alloy blocks prepared under different LPBF processing parameters.**

The Design of Q-learning Framework

**State Space:**

The state space is composed of discrete laser power (P) and scanning speed (v) values.

The laser power (P) ranges from 150 W to 350 W with a step size of 10 W, resulting in 21 distinct power values (P = {150, 160, ..., 350}).

The scanning speed (v) ranges from 800 mm/s to 1300 mm/s with a step size of 20 mm/s, resulting in 26 distinct speed values (v = {800, 820, ..., 1300}).

Thus, the dimension of the state space is 21×26=546.

**Action Space:**

The action space consists of 8 combinations of increasing or decreasing power and speed, specifically:

(10, 0) # Increase power

(-10, 0) # Decrease power

(0, 20) # Increase speed

(0, -20) # Decrease speed

(10, 20) # Increase power and speed

(10, -20) # Increase power, decrease speed

(-10, 20) # Decrease power, increase speed

(-10, -20) # Decrease power and speed

These actions affect the current power and speed, leading to transitions within the state space.

**Reward Function:**

The reward function is used to calculate the reward based on the predicted density and hardness.

If the predicted density is ≥8.20 and hardness is ≥370, the reward is 100 (target fully met).

If the predicted density is ≥8.0 and hardness is ≥360, the reward is 20 (partially meeting the target).

Otherwise, the reward is -1 (target not met).

**Q-value Update and Learning Process**

The learning process of the Q-learning algorithm is as follows:

Learning rate (α): Controls the balance between the current Q-value and new experiences (rewards and future Q-values) during each update. Set to 0.1.

Discount factor (γ): Controls the degree to which future rewards are discounted. Set to 0.9, meaning future rewards still have a high impact on current decisions.

Exploration-exploitation trade-off parameter (ϵ): Controls the balance between exploration (randomly selecting actions) and exploitation (selecting the optimal action from the current Q-table) during the learning process. Initially set to 0.9, and decays to 0.995 of its original value after each training episode, reducing exploration and increasing the probability of using the optimal strategy.

Training episodes: The total number of training episodes for the algorithm. Set to 100, indicating that the algorithm iterates up to 100 times in each training session.

In each training episode, the agent (laser) starts from a random state and performs up to 100 steps (each step selects an action and updates the Q-table based on the reward).

**Q-value update formula:**

$$\text{Q}_{\text{new}}\text{(}\text{s}_{\text{t}}\text{,}\text{a}_{\text{t}}\text{)=(1−}\text{α}\text{)⋅}\text{Q}\text{(}\text{s}_{\text{t}}\text{,}\text{a}_{\text{t}}\text{)+}\text{α}\text{⋅(}\text{r}_{\text{t}}\text{+}\text{γ}\text{⋅}\max_{\text{a}}\text{ }\text{Q}\text{(}\text{s}_{\text{t}\text{+1}}\text{,}\text{a}\text{))}$$

where:

- α=0.1 is the learning rate
- γ=0.9 is the discount factor
- *r_t_* is the current reward
- $\max_{\text{a}}\text{ }\text{Q}\text{(}\text{s}_{\text{t}\text{+1}}\text{,}\text{a}\text{)}$ is the maximum Q-value in the new state

Environmental Feedback and Reward Calculation

For each state-action pair, the system first calculates the following predictions for density and hardness:

**Line Density:**

Calculated using the formula: **line density**=P/V×0.09, where 0.09 is the spacing of the scanning track.

**Volumetric Density:**

Calculated using the formula: **volumetric density** = P/V×0.09×0.03, where 0.03 is the thickness of each layer.

Subsequently, the trained Random Forest models (density_model and hardness_model) are used to predict density and hardness, and rewards are calculated based on predefined conditions. The input features include P, D, V, ρ. The Mean Squared Error (MSE) is used as the criterion for selecting the optimal machine learning model for predicting density and microhardness. The results show that the Random Forest (RF) model performs best in predicting the density of printed samples, with a Root Mean Squared Error (RMSE) of 0.0313 g/cm³ on the test set. The same RF model also performs well in predicting hardness, with an RMSE of 10.9566 HV0.3, which is acceptable for samples with a microhardness above 300 HV0.3, as shown in Fig. S5.


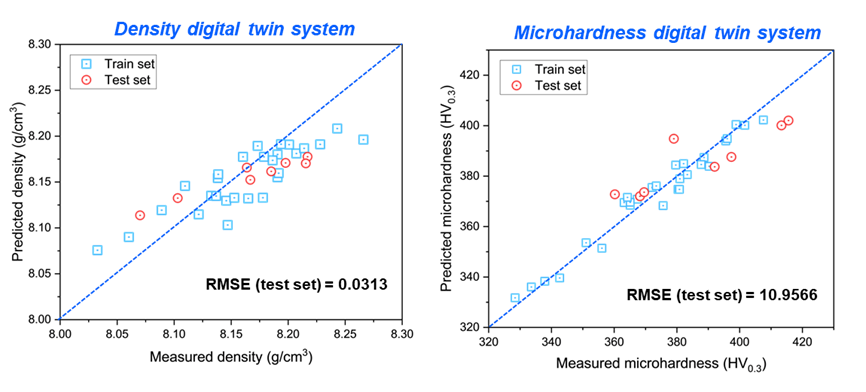


**Fig. S5 The prediction accuracy of the random forest model for density and hardness.**

**Experimental Validation**

After obtaining the optimized process parameters, selective laser melting (SLM) printing was conducted, and the density and hardness of the printed samples were measured.

To systematically evaluate the influence of thermal history on microstructural evolution and mechanical performance, two distinct heat-treatment protocols were designed and executed on the as-built alloy. The schedules are described in detail below.

1. Direct Aging (DA)
   The alloy was subjected to a single-step precipitation-hardening treatment consisting of an isothermal hold at 830 °C for 16 h, followed by still-air cooling to room temperature.
2. Complete Heat Treatment (HT)
   A two-stage solution-and-age cycle was applied. First, a supersolvus solution treatment was performed at 1180 °C for 2 h, after which the material was cooled in still air. Subsequently, the alloy underwent precipitation hardening at 830 °C for 16 h, again followed by air cooling to room temperature.

Fig. 6 presents the crack-free printed sample (Fig. 6 (a)) and electron backscatter diffraction (EBSD) characterization results of the AMN01 alloy in different heat treatment conditions, including inverse pole figure (IPF) maps, kernel average misorientation (KAM) distributions, and grain orientation spread (GOS) maps. The as-built (AB) alloy exhibits a microstructure predominantly composed of curved columnar grains, with a minor fraction of equiaxed grains observed locally (Fig. 6 (b)). This distinctive morphology arises from the complex thermal gradient and heat flow distribution during rapid solidification of the melt pool in the additive manufacturing process. The columnar grains grow preferentially along the direction of heat extraction but become bent due to localized thermal stresses. In contrast, the formation of equiaxed grains is associated with fluctuations in solidification rates and instability of the solid–liquid interface within the melt pool. Following DA treatment, the grain morphology remains largely unchanged, and the columnar grain structure inherited from the as-built state is still clearly retained (Fig. 6 (d)), indicating that this heat treatment does not significantly alter the original microstructure. In contrast, after solution treatment followed by aging (HT), the alloy undergoes pronounced static recrystallization. As shown in Fig. 6 (g), the HT condition displays a more homogeneous and equiaxed grain structure with well-defined grain boundaries and a broader grain size distribution. This microstructural evolution is attributed to enhanced atomic diffusion at elevated temperatures, which facilitates lattice rearrangement and the nucleation and growth of new strain-free grains.

Quantitative analysis of grain size distributions (Fig. 6 (j)) reveals that the mean equivalent circular diameter is 10.1 μm for the AB condition and 10.2 μm for the DA condition, whereas it increases to 13.2 μm after HT, suggesting moderate grain coarsening induced by solution annealing. Concurrently, the KAM values decrease significantly upon HT (Fig. 6 (k)), dropping from 0.83° in the AB state to 0.62° in the HT state. Since KAM reflects the degree of local lattice distortion, this reduction indicates effective relief of residual stress accumulated during printing, a decrease in dislocation density, and improved crystalline integrity [15]. Furthermore, the GOS maps (Figs. 6 (f, i)) show that the HT sample is dominated by regions with low orientation spread, with 87.3% of the area exhibiting low GOS values (typically rendered in blue), consistent with the presence of newly formed, strain-free recrystallized grains. In comparison, the DA condition retains 44.1% sub-structure regions with higher GOS values (Fig. 6 (l).

Collectively, these observations demonstrate that the HT treatment not only promotes microstructural homogenization through static recrystallization but also effectively mitigates the residual stresses introduced during additive manufacturing, thereby optimizing the internal microstructure of the AMN01 alloy.


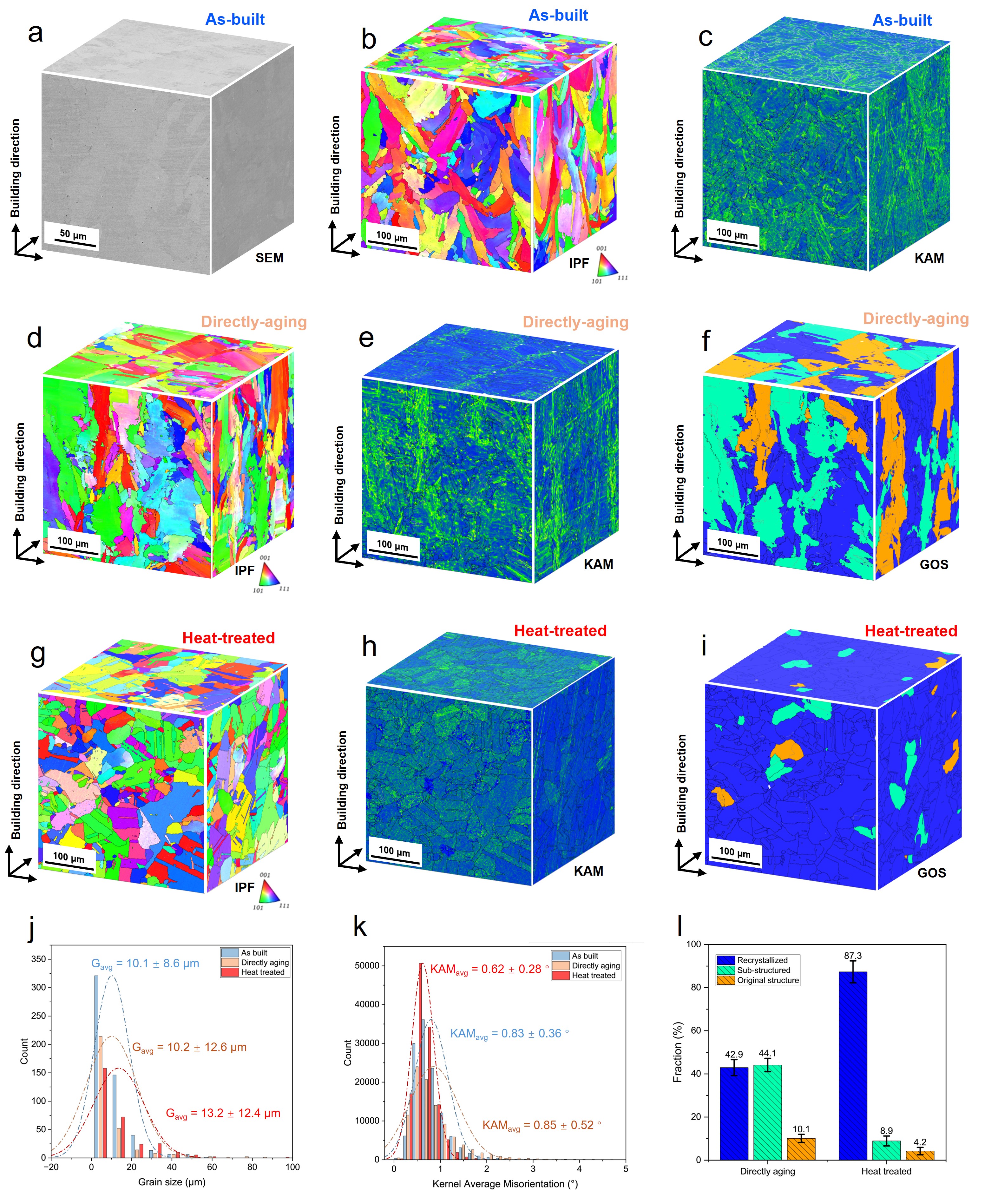


**Fig. S6 Characterization of the crack-free AMN01 alloy sample under different heat treatment conditions.** **a** SEM image of the as-built (AB) sample showing no visible cracks. **b** Inverse Pole Figure (IPF) map of the AB condition. **c** Kernel Average Misorientation (KAM) map of the AB condition. **d** IPF map after direct aging (DA) **e** KAM map of the DA condition. **f** Grain orientation spread (GOS) map of DA condition. **g** IPF map following solution treatment and aging (HT). **h** KAM map of the HT condition. **i** GOS map of the HT condition. **j** Grain size distribution of different states. **k** KAM distribution of different states. **l** GOS distribution of different states.

Fig. S7 illustrates the microstructural characteristics of the fracture surface vicinity in DA and HT state AMN01 alloys after tensile fracture. EBSD analysis indicates that there is no significant change in grain orientation for both states post fracture. The average KAM value of DA state samples is higher (Fig. 8 (c)), reflecting stronger local lattice distortion and strain concentration, consistent with its high yield strength but lower elongation mechanical behavior. In the HT state sample, the proportion of Σ3 grain boundaries near the fracture surface reaches 54% (Fig. 8 (e)). Most of these exhibit a straight and traversing morphology within grains and are associated with low GOS values (Fig. 8 (f)), characteristic of annealing twins. However, in regions of localized high strain, Σ3 interfaces arranged in bundles terminating at grain boundaries can also be identified, corresponding to higher GOS values in those grains, suggesting the presence of deformation twins [16, 17].


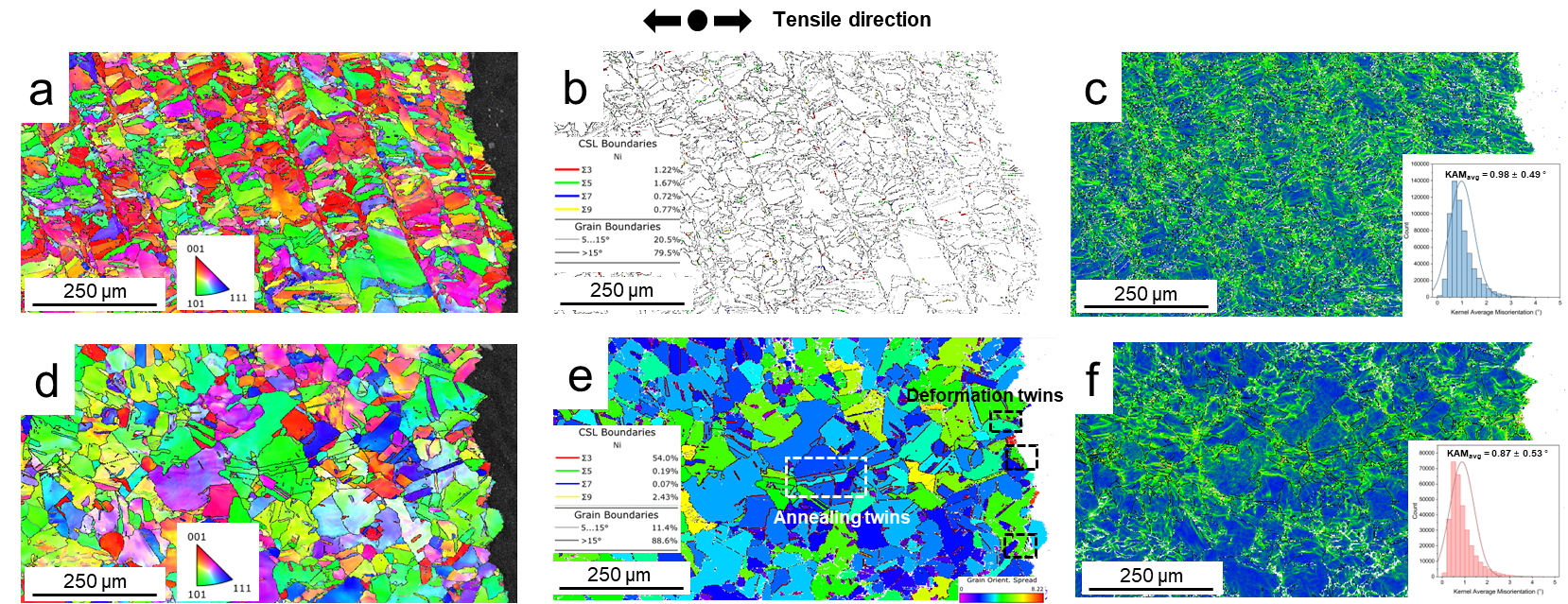


**Fig. S7 Microstructural characterization near the fracture surface of tensile-tested DA and HT AMN01 alloys. a–c** EBSD results of the DA condition: **a** inverse pole figure (IPF) map. **b** coincident site lattice (CSL) boundary map. **c** kernel average misorientation map. **d–f** Corresponding EBSD results of the HT condition: **d** IPF map. **e** CSL map (Σ3 boundaries account for 54.1%). **f** KAM map.

Fig. S8 presents the phase diagram of the AMN01 alloy calculated using thermodynamic software. The γ' phase solvus temperature of AMN01 alloy is 1110°C. The selection of 1180°C for a two-hour solid solution treatment is based on the following stringent microstructural control requirements: Establishing a super-solvus window: 1180°C provides a thermodynamic superheat of approximately 70°C above the γ' solvus line. This adequate super-solvus window ensures that the initially formed or coarse γ' phases, which may precipitate unevenly in the LPBF-as-built state, are completely and thoroughly dissolved into the γ matrix, laying the foundation for the subsequent aging stage to form uniform, fine γ' phases. The extremely high cooling rates inherent to additive manufacturing inevitably lead to significant dendritic microsegregation. The high temperature of 1180°C significantly enhances the diffusion kinetics of slow-diffusing refractory elements (e.g., W, Ta, Mo), effectively achieving complete homogenization of the alloy elements. Additionally, as shown in the full thermodynamic diagram (Fig. S8a), the onset temperature of the liquid phase (solidus line) is around 1280°C. The choice of 1180°C not only provides sufficient solid solution driving force but also maintains a wide physical safety margin relative to the solidus line, effectively preventing catastrophic local primary melting at grain boundaries.


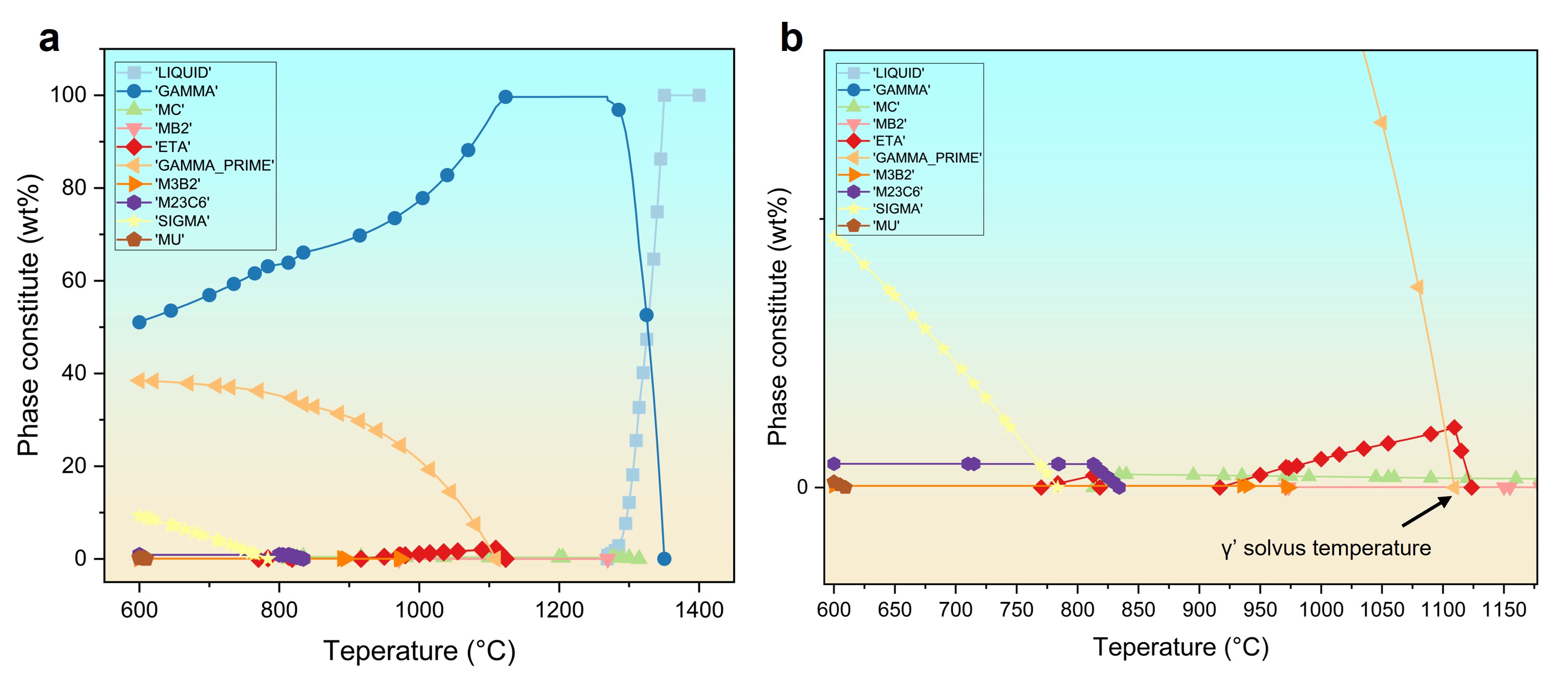


**Fig. S8 AMN01 alloy phase diagram. a** phase diagram. **b** partially enlarged phase diagram.


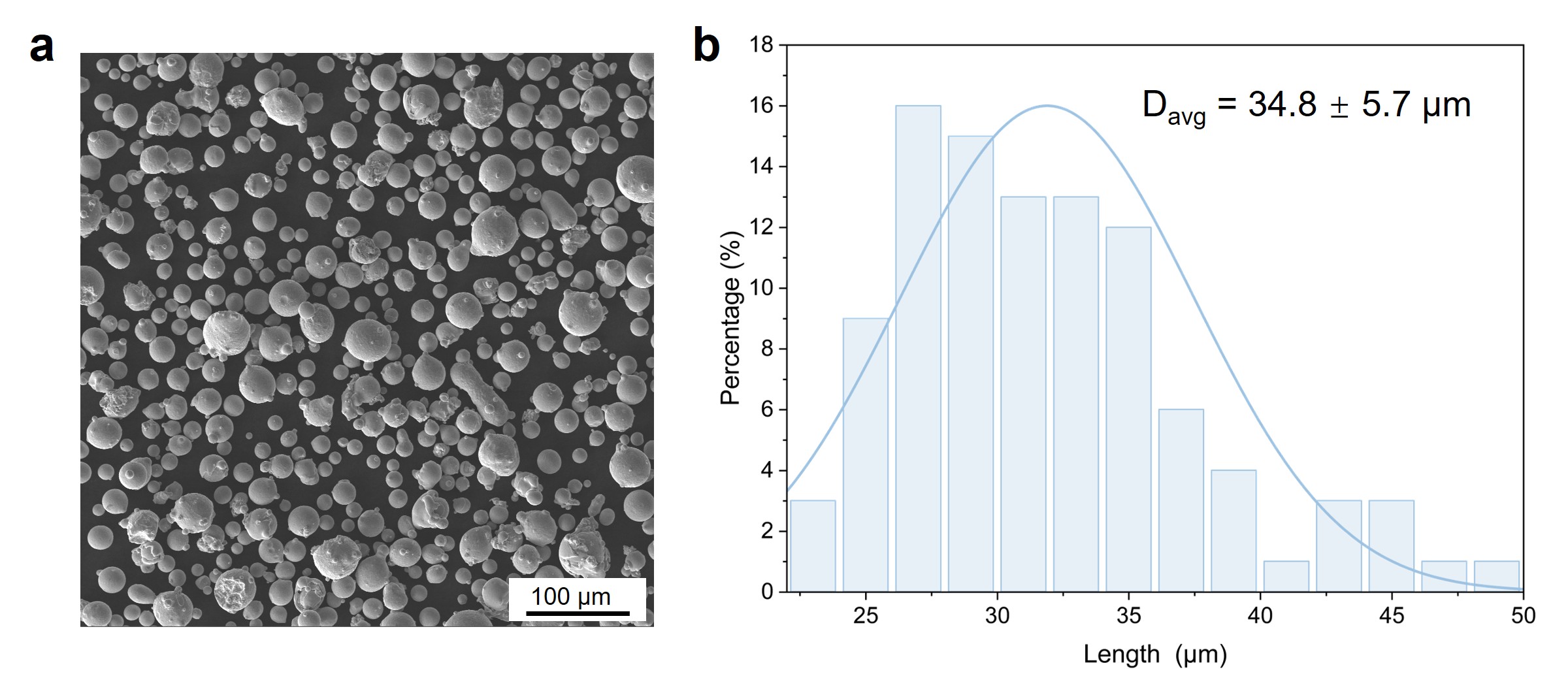


**Fig. S9 Morphology of AMN01 aerosolized powder. a** SEM image. **b** Particle size distribution.

To comprehensively understand how these descriptors influence the prediction outcomes of artificial neural network (ANN) models, the study employed Shapley additive explanation (Shap). **Fig. S10** presents the Shapley values. For instance, the Shapley means for features such as “stress”, “ΔHmix”, and “misfit” are positive, indicating their positive contribution to model predictions in most cases. It is important to note that these Shapley values are specific to this dataset and only provide interpretive significance within its data range. Overall, increased stress reduces the material’s creep fracture life; however, in the 920 dataset, higher stresses often correspond to lower test temperatures, resulting in longer creep fracture life—a phenomenon that explains the positive Shapley mean for the “stress” feature. Additionally, “ΔHmix” (mixed enthalpy), a parameter describing energy changes during component mixing, is related to material compatibility and interfacial stability and may indirectly affect creep performance. The “misfit” feature reflects lattice mismatch severity, influencing the stability and distribution of the γ′ phase, thereby impacting creep behavior. Furthermore, the features “T”, “Re”, “Mo”, and “W” all exhibit negative Shapley means. While the negative impact of the “T” feature (test temperature) on creep life is straightforward, the negative Shapley values for refractory elements such as “Re”, “Mo”, and “W” warrant further investigation. In reality, this is closely related to the compositional characteristics and operating conditions of different generations of single-crystal alloys: low-generation superalloys contain lower refractory element content and operate at lower temperatures, resulting in longer creep fracture life; whereas high-generation superalloys, despite having stricter operating requirements and increased refractory element content, may exhibit shorter creep fracture life under high temperatures and high stress conditions. This also explains the negative correlation between refractory element content and the artificial neural network (ANN) model. In summary, SHAP analysis demonstrates that during training, the ANN model did not merely extract elemental effects in isolation but rather deeply learned the complex, strongly coupled physical relationships among composition–features–test environment.

*
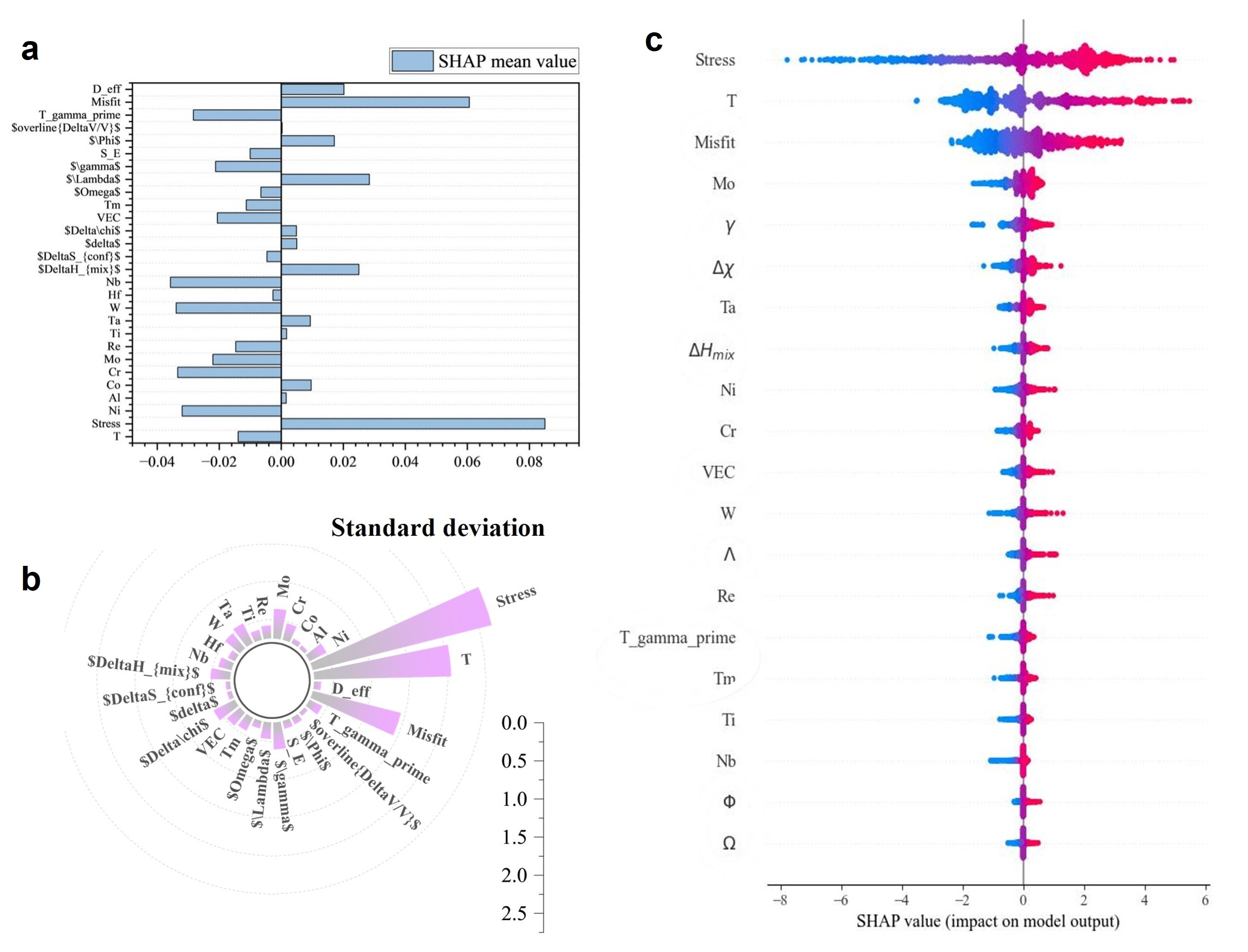
*

**Fig. S10 Analysis of feature importance and interpretability of an artificial neural network (ANN) creep life prediction model based on the SHAP (Shapley Additive Explanations) method. a** Bar chart showing the SHAP mean values of each thermodynamic descriptor and component feature. **b** Polar coordinate plot displaying the standard deviations of the SHAP values for each feature. **c** SHAP summary plot.


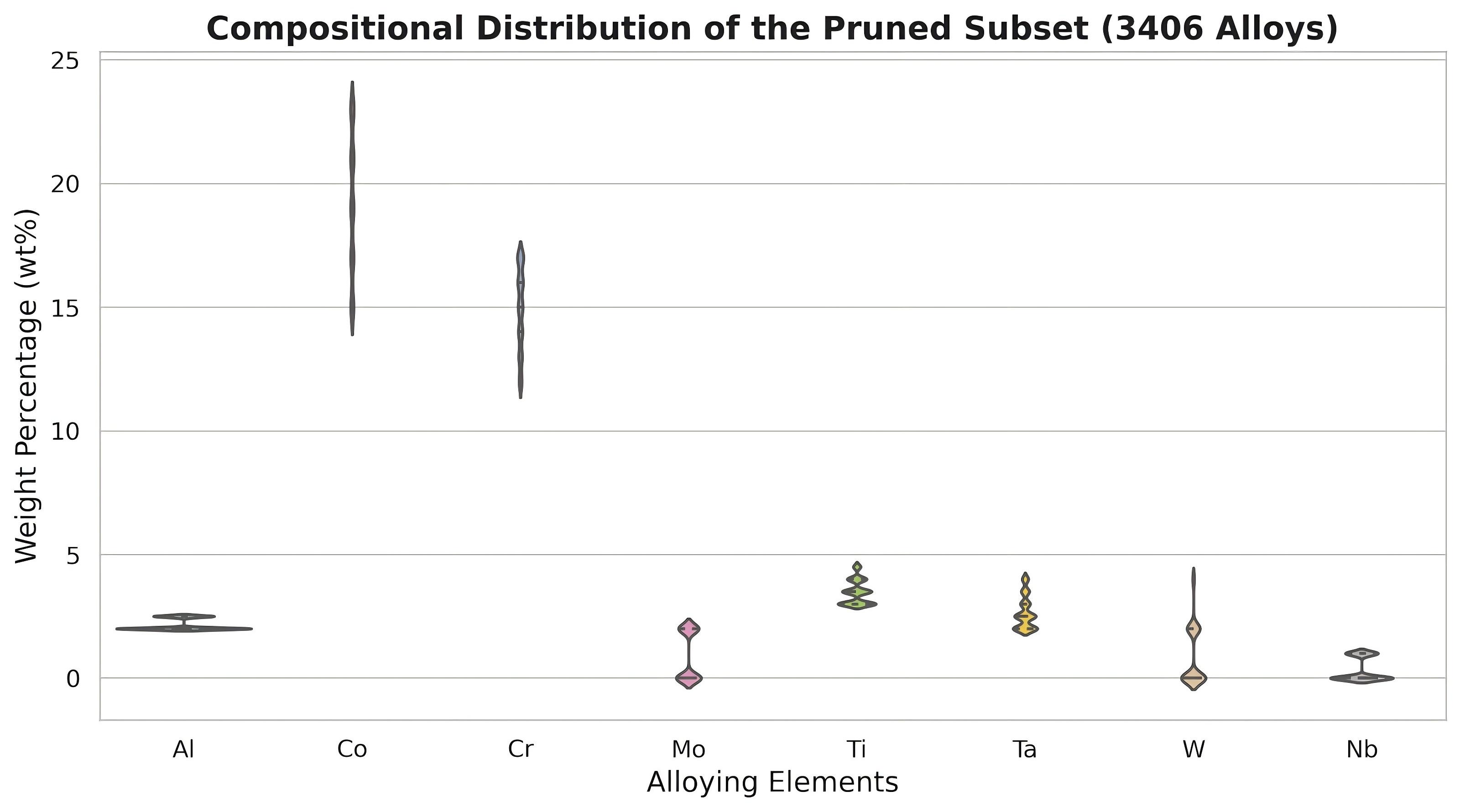


**Fig. S11 Compositional distribution of the pruned subset.**


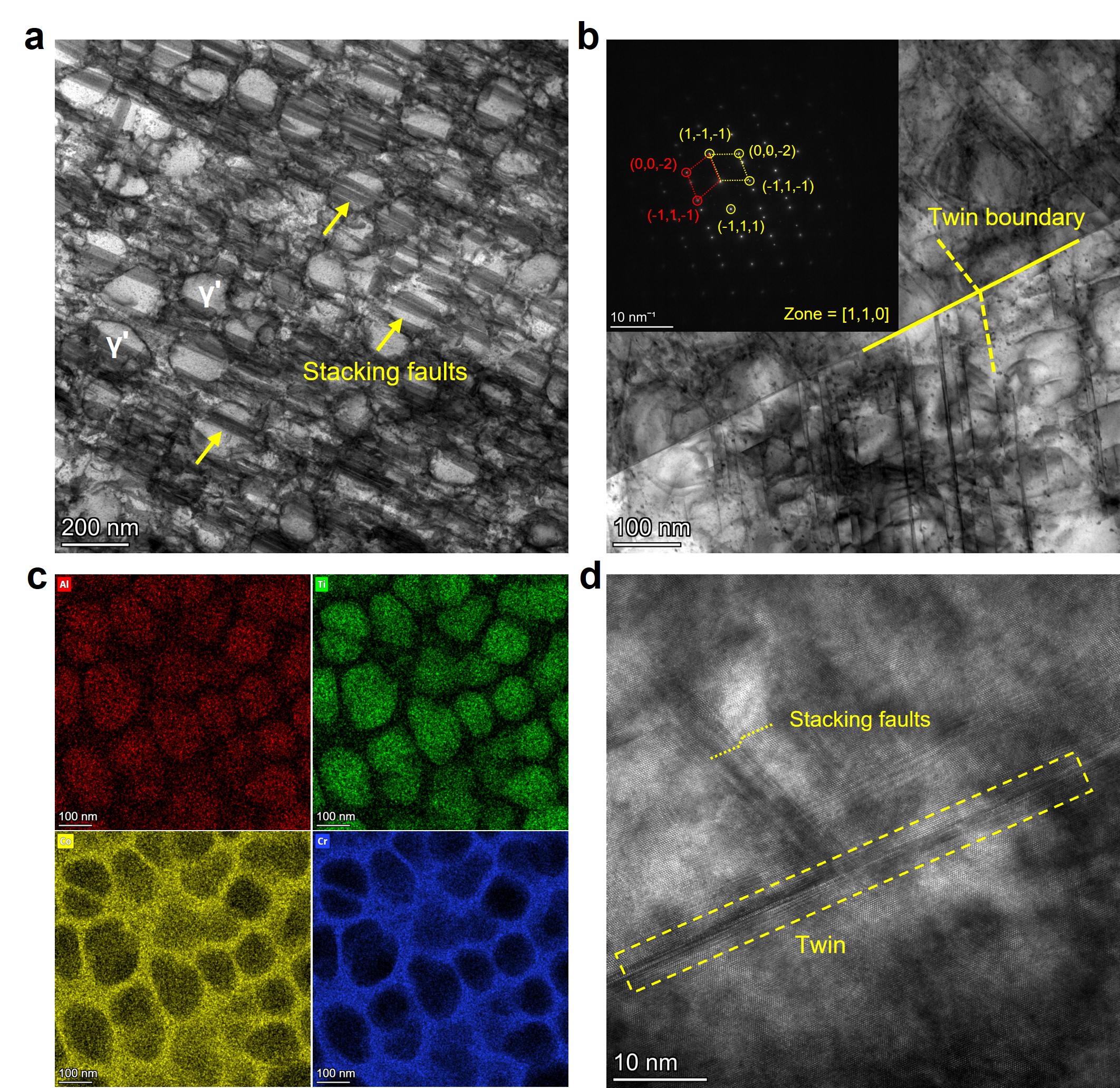


**Fig. S12 Microstructural characterization of the AMN01 alloy after creep fracture at 650°C/690 MPa. a** Bright-field TEM image. **b** TEM image revealing the formation of deformation twins. **c** TEM-EDS elemental area scan distribution. **d** High-resolution transmission electron microscopy (HRTEM) image showing atomic-scale details of stacking faults and twins.

Fig. S13 demonstrates the γ' coarsening behavior of the AMN01 alloy after long-term aging at 900°C. This behavior strictly adheres to the classical volume diffusion-controlled Ostwald ripening (LSW) theory, expressed as $\text{r}^{\text{3}}\text{−}\text{r}_{\text{0}}^{\text{3}}\text{=}\text{Kt}$, $\text{K}\text{=}\text{4.}\text{2}\text{7 ∗}\text{10}^{\text{3}}\text{ }\text{nm}^{\text{3}}\text{/}\text{h}$. After 400 hours of long-term aging, the γ' phase in the AMN01 alloy coarsens only to 241 nm, demonstrating excellent anti-coarsening performance.


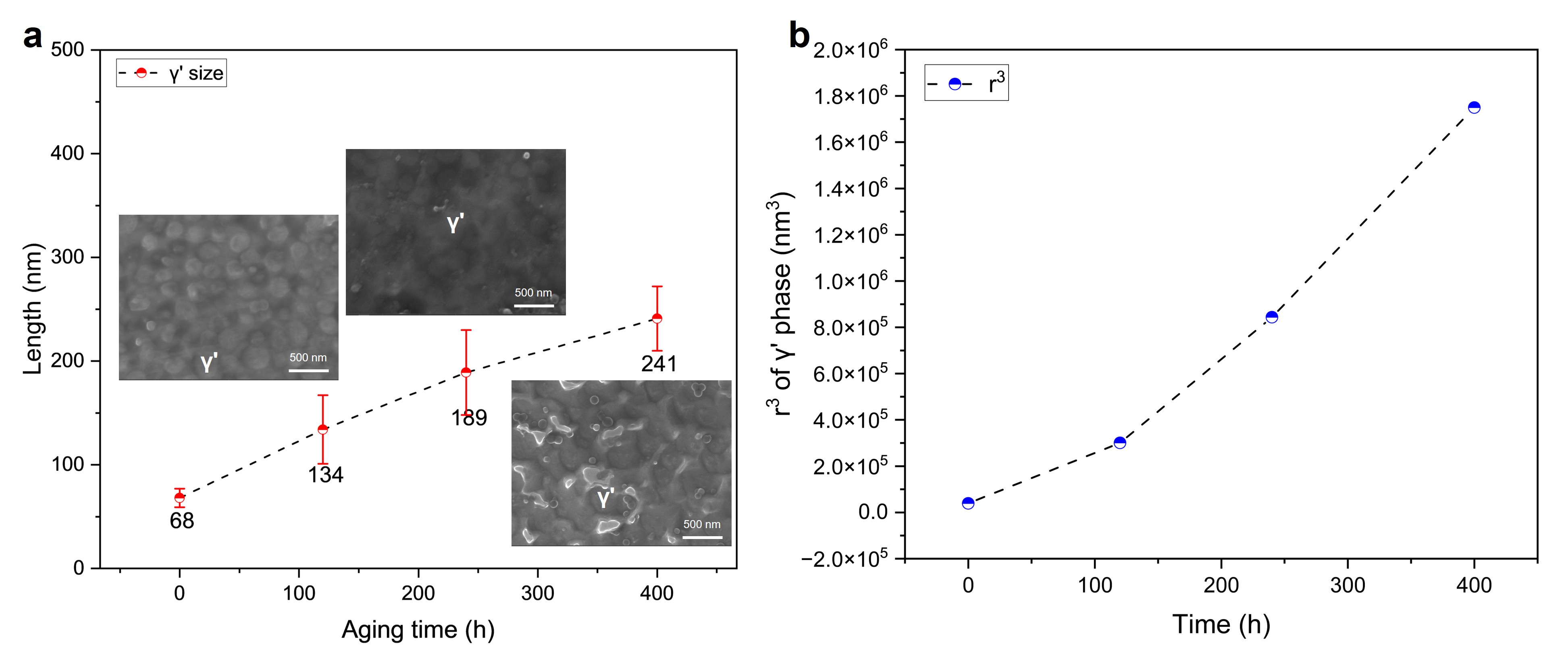


**Fig. S13 Microstructural evolution behavior after long-term aging of AMN01. a** Size of the γ' phase. **b** Cubic value of the γ' phase radius.

A fully reverse LCF test was conducted at 650°C with a total strain amplitude Δεt = 0.6%. As shown in Fig. S14, the AMN01 alloy exhibited a highly stable cyclic stress response, with peak stress exceeding 1000 MPa. Under these stringent thermomechanical coupled loads, the alloy achieved a competitive fatigue life of N_f_ = 1331 cycles. Quantitative fracture morphology analysis was performed on the failed specimens. In classical fatigue mechanics, the microcrack growth rate (*da/dN*) in the steady-state Paris region can be directly assessed by measuring the fatigue crack spacing (*s*), i.e., *da/dN* ≈ *s*. Quantitative fracture morphology analysis was conducted on the steady-state crack growth region (Stage II) of the failed specimens. High-magnification SEM images revealed clearly defined and continuous fatigue cracks with an average spacing (*s*) of 0.43 ± 0.08 μm. Based on the classical fatigue mechanism where each crack corresponds to one loading cycle, the microcrack growth rate (*da/dN*) was directly estimated as *da/dN* ≈ *s* = 0.43 ± 0.08 μm/cycle.


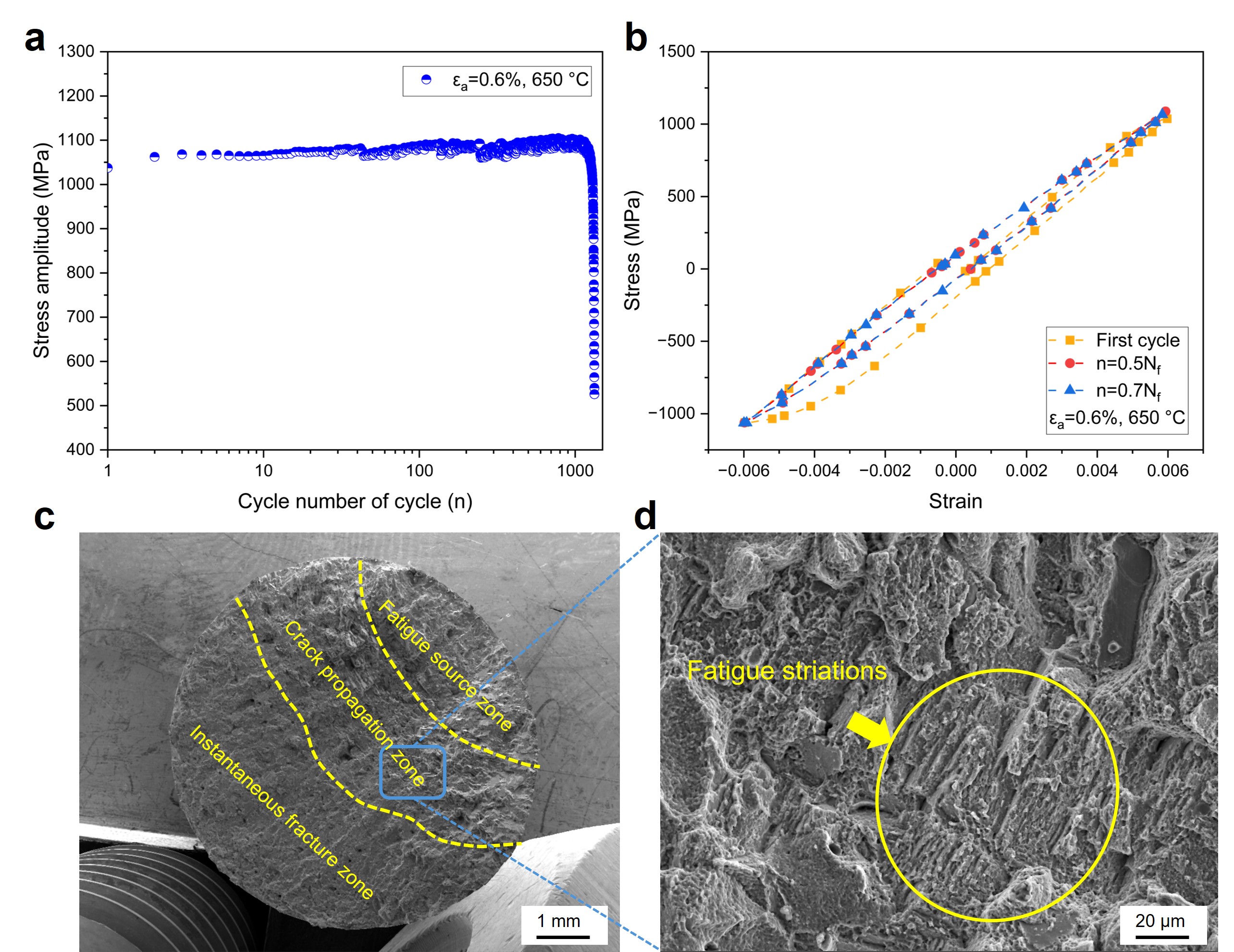


**Fig. S14 Low-cycle fatigue (LCF) behavior and fracture morphology of the AMN01 alloy at 650°C with a total strain amplitude of 0.6%. a** Cyclic stress response curve. **b** Stress-strain hysteresis loops at selected fatigue life fractions (the first cycle, 0.5 Nf, and 0.7 Nf). **c** Macroscopic SEM overview of the fracture surface. **d** High-magnification SEM image corresponding to the blue boxed region in (c), revealing characteristic fatigue striations within the crack propagation zone.

**Table S3.** **Input, prompt terms, and output examples for LLMs in component screening**

| ****Phase**** | ****Prompt Template & Interaction Logic**** | ****Output & Validation**** |
| --- | --- | --- |
| Phase 1: Knowledge Extraction | System Role: “You are an expert materials scientist. Read the provided peer-reviewed literature (e.g., Tang et al., Morinaga et al. uploaded as PDFs ^[18-23]^).”  Task: “Extract quantitative metallurgical heuristics regarding printability and phase stability for Ni-based superalloys.”  Prompt: “Identify the specific mathematical thresholds for the Al/Ti ratio, Md parameter, and Msac index to suppress hot cracking and TCP phases.” | LLM Output: Extracted explicit rules:  1. Al/Ti < 0.6  2. Md < 0.98 eV  3. M_{sac} < 4.7 wt%  Validation: Human experts cross-checked the extracted thresholds against the original literature to ensure absolute accuracy. |
| Phase 2: Code Generation for Pruning | System Role: “You are an expert Python data engineer.”  Input Context: “I have a CSV database of 76800 candidate alloys with elemental composition columns (Ni, Cr, Co, Al, Ti, etc.).”  Task: “Write a robust Python script using the pandas library to filter this dataset.”  Prompt: “Apply the exact criteria extracted in Phase 1: Filter out rows where Al/Ti < 0.6, Md < 0.98, M_{sac} < 4.7, or calculated density < 8.4. Save the high-quality subset to a new CSV file.” | LLM Output: Executable Python code (e.g., df_filtered = df[(df['Al']/df['Ti'] < 0.6) & ...]).  Validation: The 76800 candidates were pruned deterministically via the Python engine, mathematically eliminating any risk of LLM calculation errors (hallucinations). |

**Table S4.** **Input, prompt terms, and output examples for LLMs in process optimal**

| ****Phase**** | ****Prompt Template & Interaction Logic**** | ****Output & Validation**** |
| --- | --- | --- |
| Phase 3: RL Warm-Start Prior Extraction | System Role: “You are a process optimization AI for Laser Powder Bed Fusion (LPBF).”  Input Context: Provide the sparse experimental dataset (N=36) containing Laser Power, Speed, and resulting Density/Hardness.  Task: “Analyze the data trends. Identify the strict boundary for the high-risk lack-of-fusion zone and the most probable bounds for the optimal dense-hard zone.” | LLM Output: “Danger Zone: Power ≤ 190 W. Optimal Potential Zone: Power ≥ 270 W, Speed 900-1100 mm/s.”  Validation: These explicit ranges were coded directly into the initial Q-table (Q_0_) matrix. The effectiveness was quantitatively verified by comparing the search trajectory and failure rates against a Vanilla RL agent (zeros initialization). |

**References:**

1. Morinaga, M., et al., *New Phacomp and its Applications to Alloy Design.* Superalloys, 1984: p. 523-532.

2. Takeuchi, A. and A. Inoue, *Classification of Bulk Metallic Glasses by Atomic Size Difference, Heat of Mixing and Period of Constituent Elements and Its Application to Characterization of the Main Alloying Element.* Materials Transactions, 2005. **46**: p. 2817-2829.

3. Zhang, Y., et al., *Solid‐Solution Phase Formation Rules for Multi‐component Alloys.* Advanced Engineering Materials, 2008. **10**.

4. Guo, S., et al., *More than entropy in high-entropy alloys: Forming solid solutions or amorphous phase.* Intermetallics, 2013. **41**: p. 96-103.

5. Guo, S., et al., *Effect of valence electron concentration on stability of fcc or bcc phase in high entropy alloys.* Journal of Applied Physics, 2011. **109**(10).

6. Fang, S., et al., *Relationship between the widths of supercooled liquid regions and bond parameters of Mg-based bulk metallic glasses.* Journal of Non-Crystalline Solids, 2003. **321**(1): p. 120-125.

7. Yang, X. and Y. Zhang, *Prediction of high-entropy stabilized solid-solution in multi-component alloys.* Materials Chemistry and Physics, 2012. **132**(2): p. 233-238.

8. Singh, A.K., et al., *A geometrical parameter for the formation of disordered solid solutions in multi-component alloys.* Intermetallics, 2014. **53**: p. 112-119.

9. Wang, Z., et al., *Atomic-size effect and solid solubility of multicomponent alloys.* Scripta Materialia, 2015. **94**: p. 28-31.

10. Ye, Y.F., et al., *Design of high entropy alloys: A single-parameter thermodynamic rule.* Scripta Materialia, 2015. **104**: p. 53-55.

11. Seiser, B., R. Drautz, and D.G. Pettifor, *TCP phase predictions in Ni-based superalloys: Structure maps revisited.* Acta Materialia, 2011. **59**(2): p. 749-763.

12. Huang, Y., et al., *An explainable machine learning model for superalloys creep life prediction coupling with physical metallurgy models and CALPHAD.* Computational Materials Science, 2023. **227**: p. 112283.

13. Gasson, P.C., *The Superalloys: Fundamentals and Applications R. C. Reed Cambridge University Press, The Edinburgh Building, Shaftesbury Road, Cambridge, CB2 2RU, UK, 2006. 372pp. Illustrated. £80. ISBN 0-521-85904-2.* The Aeronautical Journal, 2008. **112**(1131): p. 291-291.

14. Zhu, Z., et al., *A model for the creep deformation behaviour of nickel-based single crystal superalloys.* Acta Materialia, 2012. **60**(12): p. 4888-4900.

15. Humphreys, F. and M. Hatherly, *Recrystallization of two-phase alloys.* Recrystallization and related annealing phenomena, 1995: p. 235-279.

16. Wang, Z., et al., *High stress twinning in a compositionally complex steel of very high stacking fault energy.* Nature Communications, 2022. **13**(1): p. 3598.

17. Gludovatz, B., et al., *Exceptional damage-tolerance of a medium-entropy alloy CrCoNi at cryogenic temperatures.* Nature Communications, 2016. **7**(1): p. 10602.

18. Yu, H., et al., *Computational Design of Novel Ni Superalloys with Low Crack Susceptibility for Additive Manufacturing.* Metallurgical and Materials Transactions A, 2022. **53**(6): p. 1945-1954.

19. Lippold, J.C., *Welding metallurgy and weldability*. 2014: John Wiley & Sons.

20. Kou, S., *A criterion for cracking during solidification.* Acta Materialia, 2015. **88**: p. 366-374.

21. Morinaga, M., et al., *New PHACOMP and its applications to alloy design.* Superalloys, 1984. **1984**: p. 523-532.

22. Tang, Y.T., et al., *Alloys-by-design: Application to new superalloys for additive manufacturing.* Acta Materialia, 2021. **202**: p. 417-436.

23. Carter, L.N., M.M. Attallah, and R.C. Reed, *Laser powder bed fabrication of nickel-base superalloys: influence of parameters; characterisation, quantification and mitigation of cracking.* Superalloys, 2012. **2012**(6): p. 2826-2834.
